# Planning intonation under cognitive constraints of speaking

**DOI:** 10.1371/journal.pone.0311125

**Published:** 2024-10-10

**Authors:** Nele Ots

**Affiliations:** Goethe University of Frankfurt, Frankfurt am Main, Germany; University of Missouri Columbia, UNITED STATES OF AMERICA

## Abstract

Pitch peaks tend to be higher at the beginning of longer utterances than in shorter ones (e.g., ‘The Santa is decorating the Christmas trees’ vs. ‘The Santa is decorating the Christmas tree and the window’). Given that a rise in pitch frequently occurs in response to increased mental effort, we explore the link between higher pitch at the beginning of an utterance and the cognitive demands of sentence planning for speech production. To modulate the cognitive resources available for generating a message in a visual world speech production task, the study implemented a dual-task paradigm. Participants described pictures depicting events with multiple actors. In one-half of these descriptions, the participants memorized three nouns, later recalling them and answering related questions. The results demonstrate both cognitive and linguistic influences on sentence intonation. Specifically, intonation peaks at the beginning of longer utterances were higher than in shorter ones, and they were lower under the condition of memory load than under no load. Measurements of eye gaze indicated a very short processing delay at the outset of processing the picture and the sentence, which was rapidly overcome by the start of speech. The short time frame of restricted cognitive resources thus was manifested in the lowering of the intonation peaks. These findings establish a novel link between language-related memory span and sentence intonation and warrant further study to investigate the cognitive mechanisms of the planning of intonation.

## Introduction

Spoken sentences, also called utterances, are characterized by sentence intonation, i.e., modulations of voice pitch. Modulations of pitch, or acoustically, changes in fundamental frequency (F0), are intentionally controlled (see, e.g., [1–5]) and depend on the linguistic properties of the utterance. For example, pitch peaks at the beginning of utterance are rather high when the utterance is long (e.g., ‘The fox that Sidney caught escaped sometime last night from its cage’), but rather low when the utterance is short (e.g., ‘The fox has escaped from his cage’) [6–9]. The notion of advance planning of sentence intonation proposes that the initial intonation peaks are planned ahead of the articulation because this enables the speaker to avoid dropping the voice pitch too low towards the end of the utterance [6, 8, 10]. Importantly, the lowest F0, also called intonation valley, is rather constant at the end of utterance for a given speaker [8, 10], indicating that speakers are somewhat limited in manipulating the lower register of their voice. While this physiological limitation may be an important constraint on planning utterances and intonation, it does not explain all the processes of advance planning of sentence intonation. In particular, several studies have found no effect of the length on the intonation peaks at the beginning of an utterance [4, 11, 12]. Thus, as other researchers have also noted, the study of initial intonation peaks seems to indicate that the scope of advance planning varies strongly in speakers and in different sentences [4, 8, 11, 13]. This raises the question of what other mechanisms—aside from physiological constraints—might underlie the advance planning of sentence intonation.

This study proposes and investigates the scope of (verbal) working memory (WM) in planning spontaneous utterances and intonation. It hypothesizes that the pitch peaks at the beginning of a sentence depend on the availability of mental resources in the early planning stages. To test the role of WM, this study adopts a dual-task design by combining the task of describing a picture with a recall task where speakers are asked to memorize a list of unrelated nouns before they describe the picture and to audibly recall the words after they have produced a description of the picture. In order to investigate the real-time planning of utterances, the experiment records the speakers’ eye movements during the picture description task. Therefore, this study builds on the interference effects known from the study of WM [14, 15] and uses eye-tracking methods to investigate real-time sentence production by recording eye movements during a picture description task [16–21]. The aim is to reveal the delimiting effect of the cognitive load on the planning unit and the subsequent effect of a small planning unit on sentence intonation. Altogether, the study contributes to the understanding of the relation between language production and WM, and to a cognitive account of the advance planning of sentence intonation.

### Advance planning of sentence intonation

Phonetic research on planning sentence intonation has focused on the time-related changes in F0. Specifically, research indicates that in read-aloud declarative sentences, the relative height of F0 maxima (i.e., intonation peaks) typically declines over time [7–9]. Thus, the concept of F0 declination accounts for the fact that the later an intonation peak occurs in an utterance, the lower is its height. These studies have also established that the rate of declination is slower in longer sentences than in shorter ones (e.g., ‘The fox has escaped from his cage’ vs. ‘The fox that Sidney caught escaped sometime last night from his cage’) [7–9]. Furthermore, these studies find that F0 maxima at the beginning of an utterance correlate positively with the duration of the utterance [8, 10]. Consequently, several studies have interpreted the length-dependent increase in intonation peaks at the beginning of a phrase as indicative of advance planning of the sentence intonation, as the variation in initial intonation peaks seems to accurately anticipate the duration of the entire upcoming utterance (i.e., full clauses) [6, 8, 10, 11].

The interpretation that the intonation is planned in advance of the onset of speech is further substantiated by the observation that the lowest F0 at the end of an utterance is rather consistent for a given speaker, indicating limitations in the ability to manipulate the lower register of the voice [6, 8, 10]. In terms of physiological processes, plentiful evidence shows that linguistically driven F0 maxima and minima correlate only weakly with subglottal air pressure [1–5, 22, 23]. These studies also show that laryngeal muscles effectively control linguistically driven F0 fluctuations [2, 22, 23] and work against the gradual decline of the subglottal air pressure (see also [7], pp. 88–95). Thus, while being unconscious of many changes in F0, speakers appear to control the height of the intonation peaks at the beginning of an utterance and the rate of F0 declination, preserving a comfortable pitch register for the ends of their utterances.

With the idea of advance planning of sentence intonation, phonetic theorizing seems to presume pre-speech planning units that correspond to entire utterances. In other words, the notion of advance planning of intonation suggests that speakers plan their incipient utterance as far as the end of the utterance. This presumption appears to contrast with the psycho-linguistic view of speech production, where the size of the planning unit has been a matter of debate for several decades. With some exceptions, the planning units proposed in psycho-linguistic theories are generally smaller than an entire utterance, mostly comprising the first word or the first syntactic phrase [24–34].

Indeed, while consistently demonstrating converging evidence for the advance planning of sentence intonation [6, 8, 10, 11, 13, 35–37], research also finds varying sizes of planning units in intonation planning, as the length effect on the initial intonation peaks may also be absent [4, 11, 12]. For example, in a study of Romance languages, Prieto et al. [11] observed a sporadic length effect, the occurrence of which was influenced by individual and dialectal factors among speakers. Moreover, by investigating read-aloud German, Fuchs et al. [1] found that a length-dependent increase in pitch depends on the prosodic phrasing of the spoken sentence. Namely, in their study, speakers decided to pause between subject noun phrases and verb phrases (e.g., ‘Lilli-Marlen [pause] ist eine berühmte Frau aus Suhl’, ‘Lilli Marlen [pause] is a famous woman from Suhl.’ vs ‘Lilli-Matthilda Müller [pause] ist eine berühmte Frau aus Suhl’, ‘Lilli Matthilda Müller [pause] is a famous woman from Suhl’), which in turn had a considerable effect on the correlations between the F0 maxima and the duration of the sentence. In particular, the pitch peaks correlated positively with the duration of the noun phrases at the beginning of a sentence, but not with the duration of the entire sentence. Thus, the length effect appeared to interact with the prosodic phrasing of these sentences, which, in turn, suggests that the planning units for these sentences might correspond to the first syntactic phrases rather than entire sentences, aligning with the planning units established in psycho-linguistic research [32, 33].

Given that the length effect on initial F0 peaks occurs irregularly in some contexts, languages, and dialects, it can be argued that physiological constraints on modulating a lower voice pitch are insufficient to account for all the mechanisms of the advance planning of sentence intonation. A complementary account is necessary to explain the length-dependent rise in pitch at the beginning of a long utterance. A review of the related research shows that sentence prosody, including intonation, is sensitive to mental challenges, as evidenced by various metrics, such as loudness, voice quality (e.g., creaky, breathy), speech rate, and sentence intonation [38–41]. In particular, Lively et al. [39] demonstrated that speakers tasked with tracking a dot on a computer screen while simultaneously speaking, spoke considerably louder, faster and with more monotonous intonation contours (decreased F0 variability) compared to a simple speaking task. Relatedly, Mersbergen and Payne [40] found that intonation, as indexed by the average F0, is higher when speakers are asked to read aloud the names of colours printed in contradicting ink (e.g., the word ‘black’ is printed in blue) than when they read the names of colours printed in consistent ink (the so-called Stroop task). In these multitasking contexts, the speakers needed to split their attention between planning for production and suppressing the visual signal. Thus, the resulting increase in voice pitch may indicate the high load on the attentional resources during the speaking task.

A related line of research has been interested in the effect of a load on (verbal) WM buffers on speech production. These studies usually employ dual-task settings, in which speakers are asked to describe pictures and simultaneously remember a list of words, digits or patterns of squares [14, 15, 42–45]. The dual-task settings rely on the notion of cognitive load, or the mental effort arising from splitting one’s attention between the two similar tasks, probing the particular module of WM [15, 46]. The underlying assumption is that a very similar secondary task will interfere with the main task and hinder the speakers’ linguistic performance, if the WM resources are engaged in the processing of the primary task (see also discussions in [15, 46]). Consequently, combining two attention-demanding tasks will demonstrate the involvement of attentional resources while combining two maintenance-demanding tasks indicates the utilization of WM buffers. The involvement of the verbal WM buffer in language processing for production is evidenced by the effects of interference, such as delayed speech onset latencies and increased error rates. The detrimental effect of cognitive load typically results in more incremental planning for production. This means that language chunks devised prior to articulation are relatively small, possibly consisting only of the initial segment of a sentence (e.g., ‘The Santa’ in the sentence ‘The Santa is decorating the Christmas tree’) [14, 15, 42–45].

Furthermore, the impact of cognitive load on the size of a planning unit has been shown to depend on the pre-speech planning stage. In particular, the psycho-linguistic accounts of speech production posit that language processing for speech production proceeds in three cognitive stages: conceptualization, grammatical encoding, and phonological encoding [27, 47–49]. In particular, speakers are believed to convert the intended meaning (message) into abstract-lexical representations (e.g., lemmas), assign syntactic functions appropriate for the message structure and order the constituents, given the discourse and grammatical constraints (i.e. grammatical encoding). Finally, the stage of phonological encoding activates the word forms and initiates the motor programming in terms of syllable scores for speaking. In other words, psycho-linguistic study of language production has demonstrated that before starting to speak, speakers process linguistic information in different processing stages corresponding to the levels of linguistic description of language (i.e., the phonological, morphological, syntactic and semantic levels of linguistic analysis) [27, 50–52].

Building on this research, studies investigating the role of WM in language production have shown that different pre-speech planning stages exhibit varying degrees of resistance to load manipulations. For instance, increasing cognitive load has a modest effect on the grammatical encoding stage (see, e.g., [14, 15]; but see [53]). In contrast, phonological encoding is highly sensitive to mental load, tending to become more incremental under time pressure or a shortage of mental resources [15, 25]. With regard to phonological encoding, however, Wagner et al. [45] present mixed findings; they show that the incrementality of phonological encoding increases only when WM is taxed with conceptual loads (e.g., when speakers need to memorize the size of an object, which influences the complexity of the subsequent sentences), but not when taxed with verbal loads (e.g., remembering a list of digits). This suggests that the primary influence on smaller phonological planning increments might not stem from the phonological encoding stage itself but rather from the conceptualization stage. Overall, the dual-task experiments on language production currently indicate that the stage of grammatical processing does not significantly rely on working memory resources, while phonological processing does (see also [54]). The stage of conceptual processing is extremely difficult to access, and therefore, only a few studies have addressed the resources shared between working memory and message generation [45, 53]. Generally, the view is shared that the conceptual processing is one of the most memory-demanding cognitive processes in speech planning [27, 53–55].

#### The present study

The present study proposes that the raising of intonation peaks at the beginning of utterances is a physiological response to the increased load on WM during pre-speech planning. Specifically, when speakers plan their utterances far ahead, the planning increment buffered in the WM prior to articulation needs to be large, consuming a large amount of verbal WM buffer. Drawing on the findings from multitasking [38–41], we propose that the increase in F0 at the beginning of an utterance occurs due to the cognitive system’s experiencing limits in memory buffers. Given the proposal, the increase in initial intonation peaks in long utterances is expected to occur only when the pre-speech planning increment is large, because a large planning increment imposes a load on the WM buffers. When, nevertheless, speakers devise a small planning increment irrespective of the length of the utterance prior to articulation, then the mental load remains low and length-dependent raising does not occur. This prediction aligns with the idea in [56] suggesting that intonation peaks at the beginning of an utterance may indicate the size of the planning unit devised before the initiation of articulation. Petrone et al. [56] observed that intonation peaks were lower for speakers with smaller memory spans (as measured by the reading span task) compared to speakers with larger memory spans, and took this for evidence that speakers with smaller memory spans plan in smaller units, which, in turn, explains lower pitch values.

The present study further explores this idea by experimentally controlling the available WM buffer using the dual-task methodology [14, 15, 42–45]. To address the research gap between conceptual processing and cognitive load, the present study also investigates whether the scope of verbal WM modulates the size of the planning increment at the stage of conceptualization. To determine the impact of WM on the conceptualization of messages, this study employs the visual world paradigm to investigate real-time speech planning and experimentally manipulates the degree of cognitive load using a dual-task design. If conceptual processing during speech planning requires WM resources, the conceptual planning increment should become smaller under high cognitive load, and the length-dependent pitch raising at the beginning of utterances should diminish or disappear.

To summarize, the aim is to investigate the impact of increasing mental load and the size of the conceptual planning increment on the length-dependent raising of the intonation peaks at the beginning of an utterance. The proposal is that the length effect on the intonation peaks at the beginning of an utterance indicates mental effort, which occurs only when the conceptual planning increment is large, that is, the conceptual or higher-level planning processes are weakly incremental or even non-incremental. By employing the dual-task design, this study aims to enforce small planning increments at the stage of message conceptualization, which is incremental conceptual processing. In addition, the study adopts the visual world speech production task because this method is likely to be more sensitive in capturing the earliest planning stages and quantifying the impact of memory load at the stage of conceptual processing. The experiment was approved by the Ethics Commission at the University of Tartu (3271T-27) and the design and analysis methods were registered with *PLOS ONE* [57] prior to data collection.

## Method

To verify the proposed cognitive account of the advance planning of sentence intonation, an eye-tracking experiment, consisting of two blocks, was devised. The aim of the first block was to establish the tonal differences between short and long utterances when no additional cognitive load was imposed. For this block, participants were asked to describe monochromatic line drawings depicting an action involving three actors (see Fig 2 in the Materials section, for similar designs see [58]). Either one or two of the three actors could function as the initiator(s) or the recipient(s) of an action, i.e., agent or patient, respectively. In other words, either role could be executed by one or multiple actors. In the case of multiple actors, the two actors did or did not share the identity (e.g., both are Santa Clauses or one is a Santa Clause and the other is an elf).

The concrete task setting ensured that the picture descriptions varied between short and long (e.g., ‘The Santa is decorating the Christmas trees’ vs ‘The Santa is decorating the Christmas tree and the window’). As the length effect is sometimes reported to occur between the sentences consisting of short or long subject noun phrases at the beginning of a sentence (e.g., ‘*The Santas* are decorating the Christmas tree’ vs ‘*The Santa and the elf* are decorating the Christmas tree’), but to be absent between sentences containing short or long object noun phrases at the end of a sentence (e.g., ‘The Santa is decorating *the Christmas trees*’ vs ‘The Santa is decorating *the Christmas tree and the window*’) [4], the experiment controlled for the length of the utterance at both of these sentential positions. However, the methodological assumption is that the conceptual planning processes do not differ between the two conditions of multiple actors (two agents vs two patients) because the complex visual scenes are similar in that they all require an early higher-level conceptual decision for encoding the relationships between the actors depicted [45, 58].

For the investigation of the scope of the WM, the second block of the eye-tracking experiment followed the standard dual-task design, in which speakers were asked to memorize a list of words and after a primary task, to audibly recall them [14, 15, 25, 42, 44, 59]. The picture description task served as the primary task and was embedded into the secondary word recall task. Before seeing a picture, the speakers were shown three nouns semantically unrelated to the action and the event participants in the picture and asked to remember these words (e.g., in Estonian: *traditsioon*, ‘tradition’, *otsing*, ‘search’, *investor*, ‘investor’). The conceptual load was tested by the so-called verb probe task, which means that after describing the picture, the participants were prompted with a verb, for which they were requested to orally recall the three nouns and to indicate whether each of these nouns can form a sentence with the verb on the screen. For example, the nouns ‘tradition’, ‘search’ and ‘investor’ co-occur and are acceptable with a transitive verb ‘continue’ in Estonian (according to the frequency counts in the National Estonian Corpus and the previous norming study). Crucially, the verb probe task is expected to tap the conceptual retention (see procedures in [42] for the modification of the standard WM task to tap the phonological and semantic retention) and worsen the conceptual processing in such a way that speakers spare time on making the higher-level conceptual decisions and proceed with describing the pictures in a more incremental manner. This means that instead of previewing and conceptualizing all actors in the pictures, speakers will preview and conceptualize only the actors they mention first in their descriptions of the picture.

Contrary to earlier investigations [14, 15], the experiment is designed to elicit spontaneous speech, because most of the research on F0 declination relies on read-aloud speech [4, 6, 8, 11, 60, 61]. This means that in those studies, the speakers usually read aloud written sentences. The fact that the length effect on F0 has been observed in reading only, causes Levelt [27] to cast doubt on advance planning of sentence intonation and the causal relationship between phrase length and phrasal pitch. Some recent studies have addressed this issue by specifically investigating spontaneous speech [10, 13]. Importantly, these studies examine prosodic phrases as defined by the presence of pauses (i.e., speech chunks delimited by acoustic pauses). It is notable, though, that pause-delimited speech chunks are in accordance with the notion of incremental planning, as the prosodic edge boundary can be inserted with a fairly small amount of advance planning [27]. Asu et al. [13] also do not find a constant low F0 at the ends of chunks, which underscores the incremental planning of these prosodic phrases. As the analyses of prosodic phrases extracted from the speech corpora do not record any information about the structure and the linguistic context of these utterances, specific inferences about the incrementality of planning processes cannot be made. Therefore, the findings based on the read-aloud speech or corpora of spontaneous utterances are amenable to theoretical objections.

Consequently, the present study draws on semi-spontaneously spoken Estonian because earlier corpus investigations have indicated the positive correlation between pitch peaks at the beginning of an utterance and the duration of the pause-delimited speech chunks [13]. Moreover, for Estonian, a previous visual world speech production study indicated that the intonation peaks at the beginning of an utterance vary together with the complexity and length of the content of the utterance [36]. Finally and importantly, that study also indicated that there were large planning increments for the conceptual processing stage. The present study enables the observation of whether a high cognitive load enforces smaller conceptual planning increments in Estonian.

As another contrast to existing research, earlier investigations often trained speakers to use specific lexical items (e.g., names of the pictured actors learned before the experiment) [14, 15]. Conceptual processing, however, can most readily occur in speakers who have not yet encountered the concepts and words of interest in the context of a particular experiment. Thus, to examine the most natural conceptual planning processes, the experiment elicits spontaneous utterances in response to pictures. Spontaneous speech production entails large segmental and lexical variation between these sentences, which makes the use of latencies in the onset of speech as standard approximations for cognitive effort less reliable. Moreover, the earlier findings suggest that the stage of conceptual processing involves only about 30% of the time from the picture presentation until the start of speech [17–19, 62]. Therefore, the time between the onset of the picture stage and the start of an utterance subsumes different stages of planning for production. In turn, the measure of the latency of the onset of speech may prove to be somewhat insensitive for diagnosing the interruptions and interferences of cognitive load during pre-speech conceptual processing.

The experiment records speech time-locked to the eye movements in a picture description task to overcome the limitations of measuring the latency in the onset of speech. Past research has established this method as reliable for assessing real-time language processing and planning for production [17, 19, 20, 62]. Importantly, many studies since Griffin and Bock [17] have established that some of the stages of utterance planning are clearly observable in the location to which the eye is directed while describing a picture [16–21, 62, 63]. Namely, the picture naming studies have observed that before naming an object, subjects look at an object for a long duration to recall its name, indicating lexical and possibly syntactic processing (e.g., [49]). Similar long viewing times also occur while describing pictures in full sentences. Nevertheless, these long viewing times in picture-description tasks appear much later than in picture-naming tasks (e.g., approximately 1000 ms from the picture onset). In particular, the visual attention in the first 0 to 400/600 ms of being presented with a picture is split between the different regions of the depicted actors to be mentioned in subsequent utterances. Only in later time windows of the picture processing does the visual attention concentrate on one of the actors for a longer period. Griffin and Bock [17] argue that the early time window of divided visual attention provides a conceptual frame that guides the subsequent rapid processing of the actors in the order they are mentioned by a subsequent utterance. In other words, the eye movements within this early time window of planning index the conceptual processing of incipient utterances.

Importantly, research shows that the proportions of eye fixations varying as a function of time in this early time window of picture processing are highly sensitive to the linguistic features of the subsequent utterances. For instance, the proportions of these early eye fixations appear to depend on the word order [20, 62], the presence of morphological case-marking [21], syntactic priming [18, 64] and lexical diversity [18]. For example, in a study with Dutch speakers, Konopka and Meyer [18] observed that conceptualization proceeded more or less incrementally depending on how accessible the name of the first-mentioned actor was. In particular, they found that when the name was easily accessible, speakers “fell back” on a strictly incremental, that is, word-by-word or concept-by-concept planning strategy. This was indicated by the greater proportions of eye fixations on the first-mentioned actors at around 400 ms after the presentation of the picture. Thus, in the earliest timeframe of processing the picture, the increase of the proportions of fixations on the first-mentioned actors is taken to indicate more incremental conceptual processing, whereas the decrease of the these proportions is taken to indicate less incremental conceptual processing [17, 18, 20, 21, 62, 64]. Consequently, the early and very brief apprehension-phase at the outset of the linguistic encoding of the visual content is linguistically driven. Furthermore, the eye movements recorded in this early time frame of processing the picture approximate the degree of incrementality of the conceptual processing during the real-time planning and production of an utterance. As such, eye movements constitute an established behavioural measure and a proxy for cognitive processes involved in language processing for production.

### Hypotheses

For pre-speech planning, I assume relatively large planning increments for the picture descriptions uttered in response to complex visual scenes. Research indicates that the increment of planning for active sentences with an agent-before-patient structure (subject-verb-object ordering) can correspond to an entire sentence [15, 25, 29, 45, 58]. For instance, Oppermann et al. [29] demonstrated, for German, that planning of sentences with a frequent subject-verb-object ordering, such as ‘*Die Maus frisst den Käse*’ (in German, ‘The mouse is eating the cheese’), can be successfully executed in full even at the phonological planning stage. Similarly, in English, Ferreira and Swets [25] showed that speakers plan their active sentences non-incrementally when no time constraint is imposed. Additionally, a recent study on Estonian sentence and intonation planning indicated large conceptual planning increments for subsequent transitive sentences with complex contents (e.g., ‘The man is pulling the donkey with a basket’; [36]). Therefore, the present study assumes that conceptual planning in Estonian under a low cognitive load is only weakly incremental or even non-incremental, but is expected to become more incremental under high cognitive loads.

The effect of cognitive load on incrementality should be observable in the unequal distribution of visual attention between the first-mentioned and second-mentioned arguments of an event. For smaller conceptual planning increments, the visual focus is expected to primarily be on the first-mentioned semantic argument (i.e., the agent), with the gaze shifting to the second-mentioned semantic argument (i.e., the patient) only later during the processing of the picture. In other words, if conceptual planning processes share resources with working memory, then the increments of conceptual processing should be smaller under a high than under a low cognitive load. This means that under a high cognitive load, speakers are expected to conceptualize and articulate the agent first before conceptualizing the patient, whereas under a low cognitive load, they could conceptualize the entire sentence in one go.

Alternatively, the higher cost of the speaking task might influence the stage of linguistic encoding and especially the lexical retrieval. For this, the manipulation of the cognitive load is expected to affect the pre-speech visual processing of the agents, so that they are gazed at for longer times under a high cognitive load than when under a low cognitive load. In other words, a memory deficit is expected to manifest itself as a processing delay at the stage of linguistic planning (or lexical retrieval).

**Fig 1:**
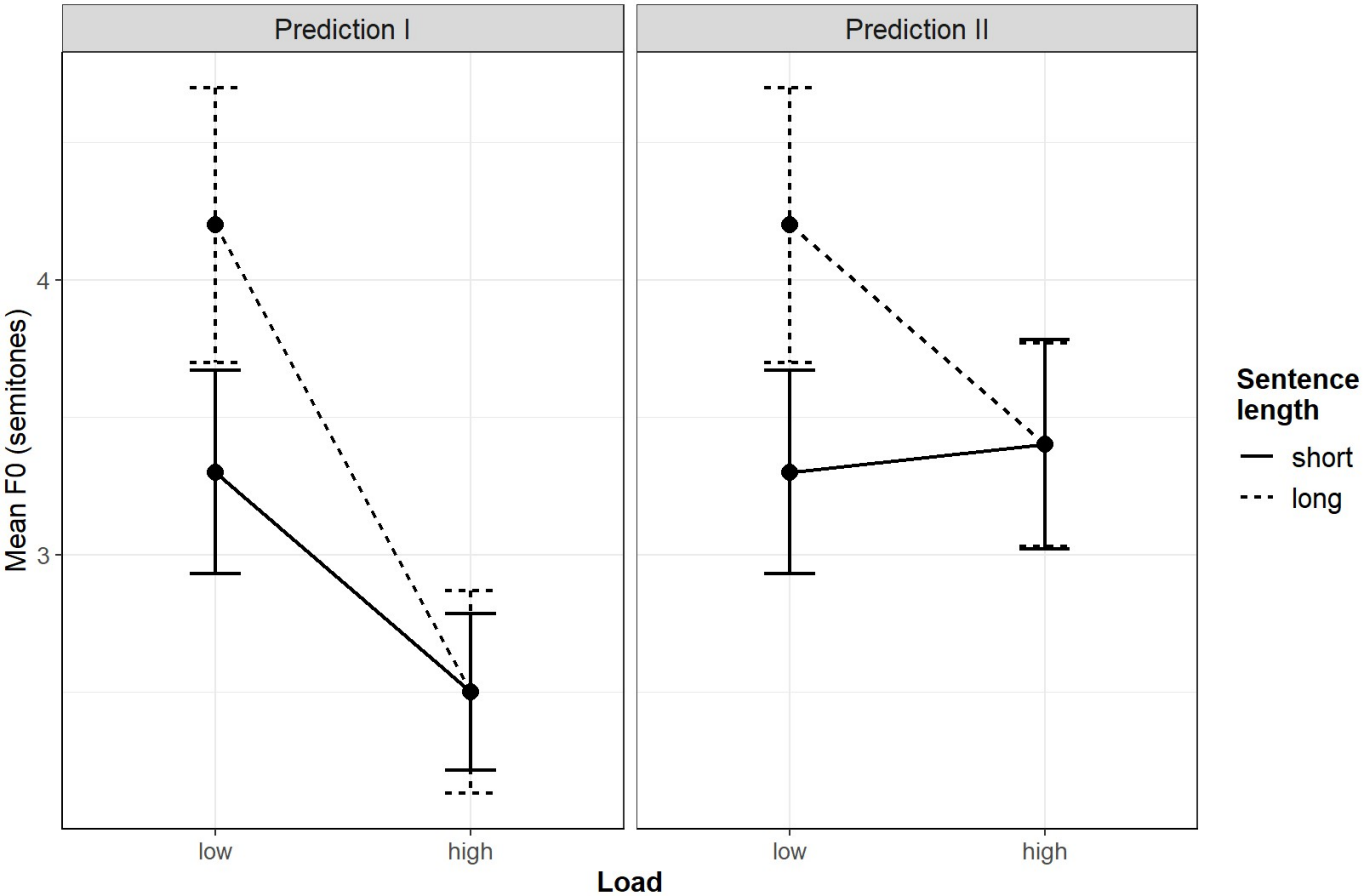
Illustration of hypotheses regarding intonation peaks at the beginning of a sentence, based on the invented data. Arrows indicate the 95% confidence intervals.

If a high cognitive load reduces the size of the planning increment compared to a low load, initial intonation peaks are expected to be higher under a low cognitive load than under a high cognitive load, in response to the smaller planning increments that consume less WM buffer. The dual-task design anticipates two possible outcomes for the initial pitch scaling. Firstly, highly incremental conceptual processing, which involves only foreseeing the mention of the first concept in subsequent utterances, is expected to correlate with lower intonation peaks. In this scenario, the intonation peaks at the beginning of an utterance are predicted to appear at their lowest level under a high cognitive load, regardless of the length of the utterance (see Prediction I in Fig 1). In other words, the scaling of intonation peaks under a high load corresponds to the durations of the very first nouns, resulting in very low intonation peaks at the beginning of an utterance.

Alternatively, a high cognitive load might have an effect on the intonation peaks at the beginning of an utterance independently of the incrementality of the conceptual planning. The load of splitting one’s attention between the two linguistic tasks might cause a rise in pitch, counteracting the lowering effect of incremental planning. In this alternative scenario, the raising effect of the cognitive load and the lowering effect of the incrementality add up in such a way that the intonation peaks have about the same height as the intonation peaks in short utterances under low cognitive loads (see Prediction II in Fig 1). In other words, the additive effects of incrementality and the high cognitive load are predicted to affect the intonation peaks in long but not in short utterances.

### Materials

The materials consisted of two types of stimuli: pictures and word lists.

#### Pictures

**Fig 2:**
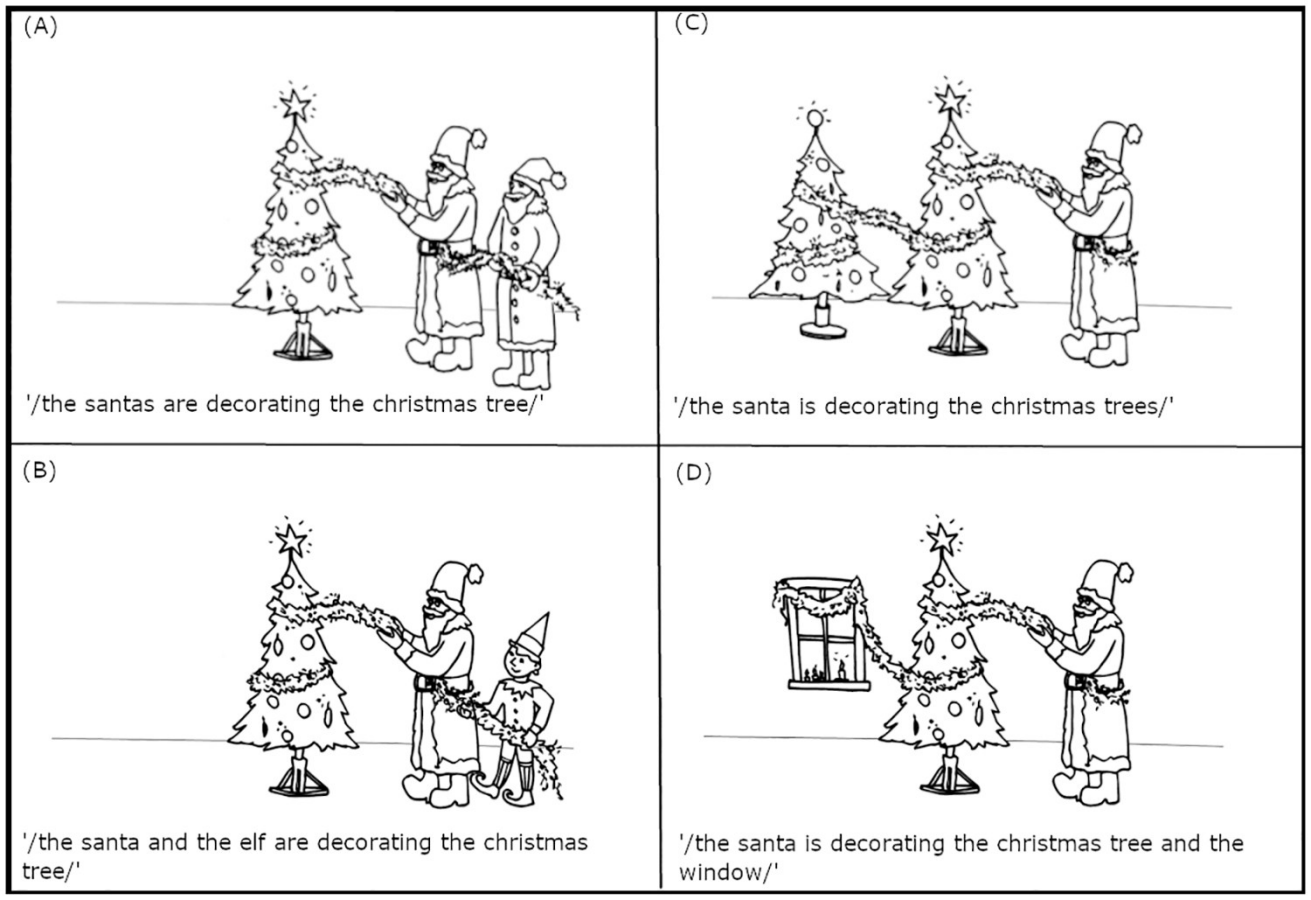
An example picture in four experimental conditions eliciting short and long sentences.

To incite utterances differing in length, speakers were asked to describe pictures of actions (i.e., transitive events) involving multiple actors. The aim of the pictorial design was to manipulate the two two-level visual factors, the so-called Double Actor and Identity. Across the four conditions, the pictures depicted three actors, two of whom shared a thematic role. The two core roles of actions typically include i) an initiator or agent, and ii) a undergoer of the action or patient. For the factor Double Actor, the two thematic roles were distributed among the three pictured actors in such a way that either two agents acted upon a single patient (Fig 2a and 2b) or a single agent acted upon two patients (Fig 2c and 2d). For the factor Identity, the two actors in the same thematic role either did or did not share their identity (e.g., they were both Christmas trees or they were the Christmas tree and a window). The two visual factors with two levels were fully crossed, resulting in four pictorially controlled conditions. In particular, the four conditions showed (i) same multiple agents acting on a single patient (e.g., Santas decorating the Christmas tree), (ii) different multiple agents acting on a single patient (e.g., the Santa and the elf decorating the Christmas tree), (iii) a single agent acting on similar multiple patients (e.g., the Santa decorating Christmas trees), or (iv) a single agent acting on different multiple patients (e.g., the Santa decorating the Christmas tree and the window).

Altogether, 64 different pictures of simple events (target trials) were created for the study (4 × 64 = 256 pictures in total). Another set of 84 pictures served as distractor trials. The distractor trials depicted a variety of transitive and intransitive events comprising one to four entities discriminable as actors of an event. Finally, a set of 12 pictures (practice trials) was assembled of 6 pictures similar to the pictures in the target pictures and of another 6 pictures similar to the pictures in the distractor trials.

#### Word lists

For the cognitive load and the conceptual retention task, one-half of the pictorial items were combined with a verb probe task. For the verb probe task, two sets of verbs were selected based on their collocation frequencies in the Estonian National Corpus [65]. The sets of transitive and intransitive verbs were restricted to participate in a moderate numbers of collocations with nouns referring either to animate (marked for subject function) or inanimate entities (morphologically marked for object function). The moderacy restriction (the co-occurrence frequency being between the Leipzig lexical frequency classes of 8 and 12 [66]) was applied for two reasons. First, when a verb participates in a small number of collocations that also occur frequently in the corpus, the verb might aid the recall of the memorized words. Second, when the verb participates in a large number of collocations, it may be difficult to delimit the set of nouns that are conventional with these verbs. While transitive verbs were selected in such a way that they were free to occur with both animate and inanimate referents, the intransitive verbs were selected to encode actions typical of only animate referents. In the memory trials, half of the target pictures were followed by transitive verbs and the other half of the target trials by intransitive verbs.

The memory lists of three nouns were constructed so that they contained a noun that referred to an animate referent and two nouns that referred to inanimate referents. Notably, the variation of animacy in the to-be-memorized word lists was regarded as the conceptual aspect of the intended message and to tap into the conceptual planning processes. The verb probe task was built on the transitivity of verbs. In particular, the lists of nouns (e.g., in Estonian: *traditsioon* ‘tradition’, *otsing* ‘search’, *investor* ‘investor’) were selected in such a way that all nouns were likely to form sentences with transitive verbs (e.g., in Estonian: *jätkama* ‘continue’) but only nouns referring to animate referents could form sentences with intransitive verbs (e.g., in Estonian: *muretsema* ‘worry’). Thus, in target trials, all nouns in the memory list were compatible with the corresponding transitive verbs but never with the intransitive verbs. The compatibility of the verb with the respective memory list was confirmed in a pre-test with a different sample of native Estonian speakers. To balance the factor of verb type, 50% of the distractor trials included nouns that were incompatible with the transitive verb prompt. The other half of the distractor trials were prompted together with intransitive verbs that were compatible with all of the nouns on a list. Due to the limitations of time and financial resources, the lists and prompts that were used in the distractor trials were selected based on the corpus statistics and were not all pre-tested.

Altogether, 32 pairs of three-word list and a noun were constructed for the target trials. For the distractor trials, another 42 pairs of three-word list and a noun were created. Finally, 12 additional pairs of a three-word list and a verb served as practice trials.

### Design

The two visual factors Double Actor and Identity, as well as the factor Cognitive Load, all having two possible levels, were fully crossed, resulting in 8 conditions in total. The two-level fixed effect Cognitive Load reflects whether the picture was described while maintaining a list of nouns in WM or not (levels low and high). The trials with no load constituted the first block of the experiment (32 target pictures) and the trials with a load (32 target pictures) constituted the second experimental block. The pictures were subdivided into the blocks in such a way that there was the least possibility for semantic overlap between the target and distractor items. The order of the pictures within a block (low vs high load) was randomized.

Four lists of target trials were created by distributing the 256 target pictures between the four conditions (Double Actor crossed with Identity) using the Latin square approach. The target pictures in the lists were distributed between the same set of 84 distractor pictures in such a way that there were one or two distractor pictures for every target picture, resulting in 148 experimental trials per list in total. Another set of four lists was created, where the leftward placement of the agents was reversed, resulting in eight lists. For the recall task, all 8 lists were reversed for a further set of 8 lists, amounting to 16 lists altogether. In all 16 lists, the second half of the trials included the word recall and verb probe task.

Participants were presented with all 8 conditions across 64 target pictures (repeated measures, within-subject, between-item). The within-subject design was necessary because F0 tends to be highly variable and affected by many other, even non-linguistic, factors. The tonal differences between the conditions can most reliably occur within a speaker, ideally also within the items of the same speaker. Due to this practical limitation, most of the classical intonation research typically records all conditions for a speaker (see, e.g., [4, 6–11, 67–70]). The within-item design is not optimal for the purposes of this study, as the aim was to investigate natural and spontaneous planning processes, which might cease after the first encounter with a pictorial item. Thus, the repeated measures design with the factors Cognitive Load, Double Actor and Identity being within-subject factors and the pictorial items being in between represented the best fit between the purposes of the study and the limitations faced by F0 measurements. In other words, the design resembles the ANOVA repeated measures design with a within-subject factors eliciting 64 measurements (8 images × 8 conditions) per participant.

### Participants

In total, 84 adult native speakers of Estonian (one genderfluid, 63 females with an average age of 24.3 years and 20 males with an average age of 28.6 years) participated in the study. All participants reported normal or corrected to normal vision (soft contact lenses included, glasses excluded), no diagnosed language impairments or hearing disabilities. All participants signed a written consent form informing them about the risks of the study and their right to stop participating in the experiment at any time without any consequences. Participants were reimbursed 5 euros for their participation in the study.

### Procedure

The speakers were recorded at the phonetics lab of the University of Tartu between 14 July and 24 September in 2021. The pictures were presented on an SR Research EyeLink 1000 Plus desktop-mounted eye tracker (sampling rate 1000 Hz, distance to participant ca. 90 cm, refresh rate of the display 60 Hz). The head was leaning on the head support but the chin was left unsupported because the study required the participants to speak. The participants were assigned to the 16 lists of experimental trials semi-randomly so that on average, each list was seen 5.25 times.

A concrete task setting ensured that the fully crossed pictorial factors Double Actor and Identity elicited short and long utterances at the two different sentential positions. In particular, the speakers were instructed to use plural noun phrases for the actors sharing an identity (e.g., ‘Christmas trees’) and coordinated noun phrases (NPs) for the actors differing in identity (e.g., ‘the Christmas tree and the window’). Consequently, utterances that included NPs were shorter than the utterances containing coordinated NPs. The factor Double Actor triggered the plural and coordinated NPs either at the beginning of the sentence (agent) or at the end of the sentence (patient) in transitive sentences.

**Fig 3:**
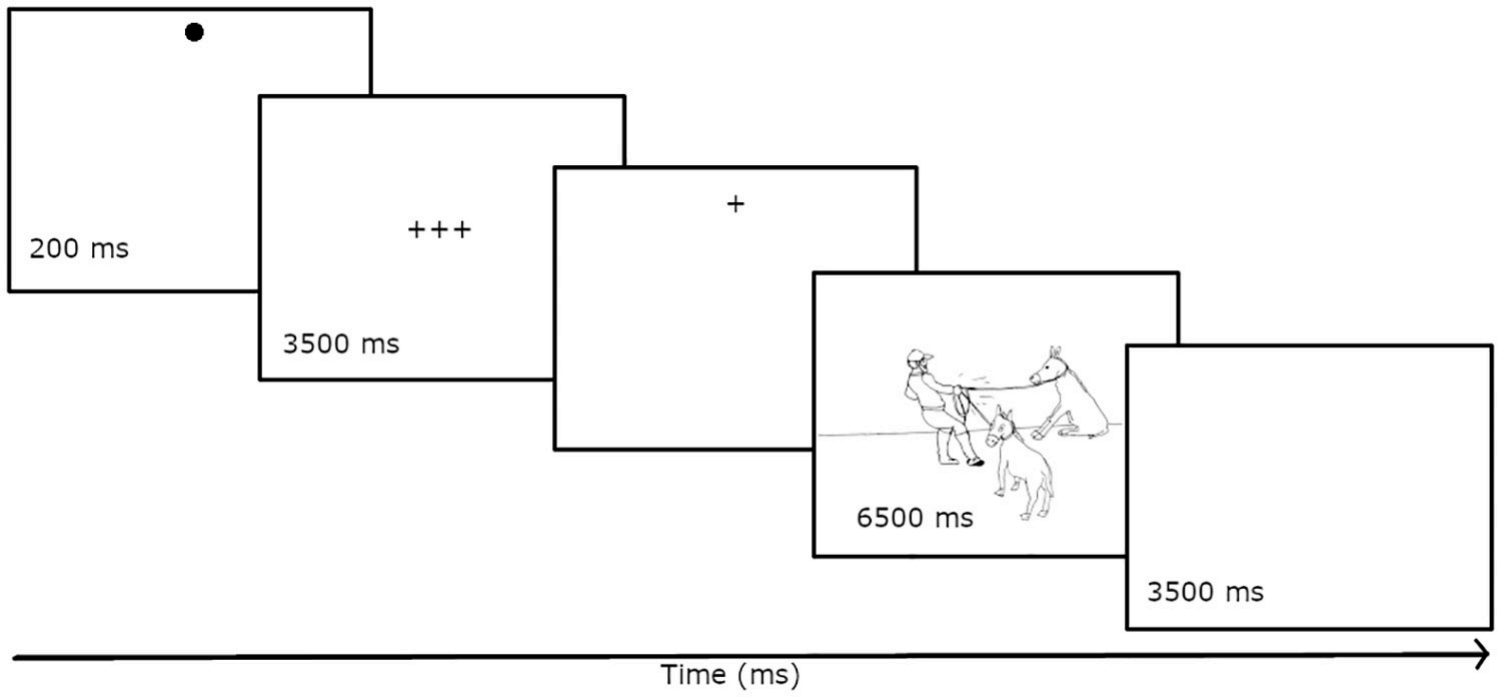
Presentation sequence of stimuli in trials without a concurrent memorization task. The first screen with a dot on top is for checking the calibration, the third screen with the cross on top triggers the presentation of the picture as soon the eye is directed to it.

**Fig 4:**
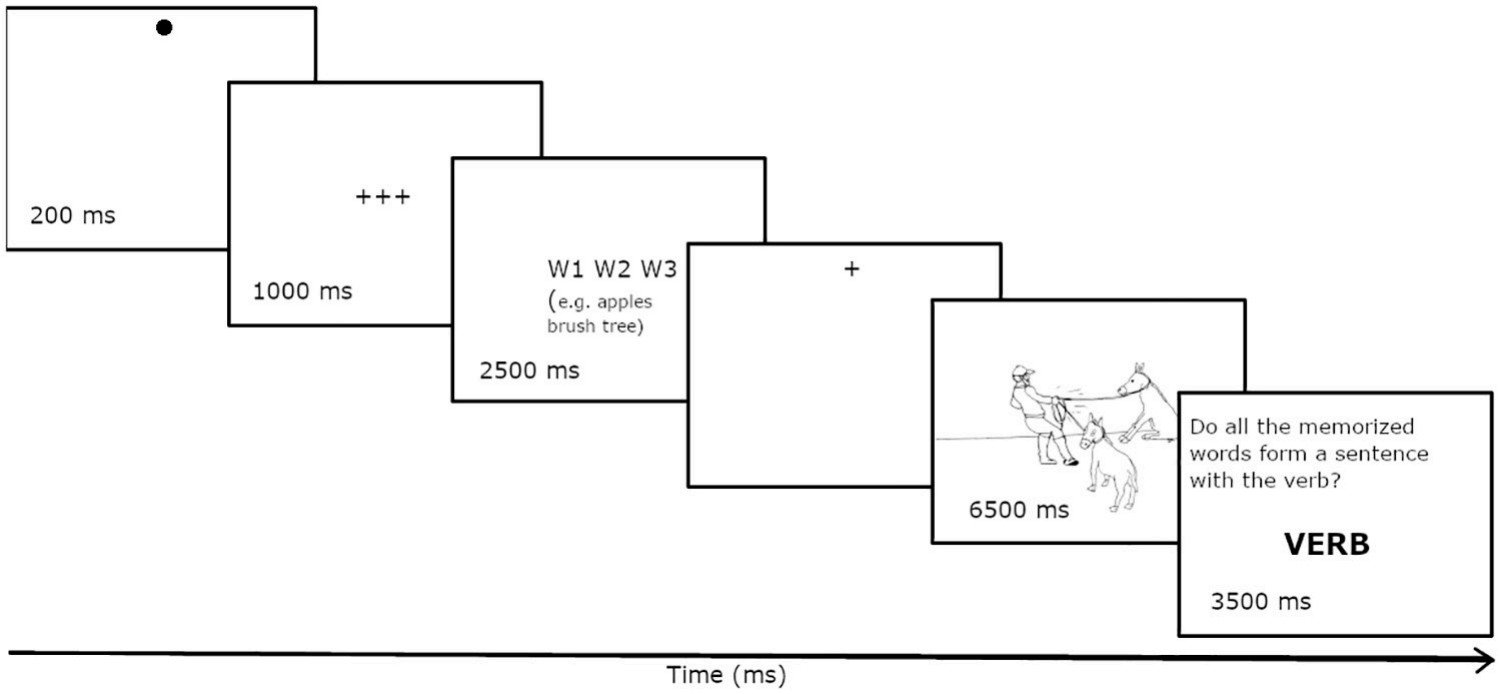
Presentation sequence of stimuli in trials with a concurrent memorization task. The first screen with a dot on top is for checking the calibration of the eye camera, the fourth screen with the cross on top triggers the presentation of a picture as soon as the eye is directed to it.

The experiment proceeded in two blocks. In the first block, the participants’ task was to describe actions by mentioning all three depicted actors using either plural or coordinated NPs. The presentation of a picture was preceded by a display of three crosses in the middle of the screen for 3500 ms (see Fig 3). This was necessary to keep the duration of trials with and without the memory load comparable. In the second block, the participants’s task was to memorize the three words, describe the picture, and then solve the verb probe task (see Fig 4). Therefore, the presentation of a picture was always preceded by three crosses for 1000 ms and three words in the middle of the screen for 2500 ms, and always followed by a display of the verb and the verb probe task. The pictures across the two blocks were presented in a way that was contingent on the gaze, and they stayed on the screen for 6500 ms (Figs 3 and 4). The participant was given another 3500 ms to finish speaking.

Before the first experimental block, the experimenter explained the picture description task by showing sample pictures together with the possible sufficient descriptions written at the bottom of the screen. This explanation was followed by six practice trials. The second experimental block started with the explanation of the verb probe task involving memorization. In particular, the participants were required to remember the words and to audibly recall them after a sufficient picture description. They were asked to indicate whether the recalled words could form sentences with the verb on the screen. If they thought so (in the case of transitive verbs), they were asked to say ‘Kõik sobivad’ (They all fit). If they thought not (in the case of intransitive verbs), they were requested to say ‘Ei sobi’ (Not all words do fit). In the target trials, a correct response to a transitive verb prompt is that all words fit with the verb on the screen. For example, the words ‘tradition’ and ‘search’ on the wordlist “*traditsioon, otsing, investor*” (in Estonian: ‘tradition’, ‘search’, ‘investor’) can be ‘continued’ (transitive verb) and an ‘investor’ can ‘continue (with)’ something. In contrast, neither ‘tradition’ nor ‘search’ can ‘worry’ (intransitive verb) but an ‘investor’ can. The memory task and the verb probe task was first rehearsed without and then with describing pictures. The second experimental block in which memorization and recall were interposed by a task of picture description started after the 6 practice trials. The participants had to take a break between the two blocks but within the blocks, they were free to take a break at any time needed. On average, the recording session took about 60 minutes of the participant’s time.

### Analysis

#### Pre-processing and data selection

All picture descriptions were manually transcribed with the help of the phonetic analysis software Praat [71]. The transcribing process included the annotation of the onset and offset times of the utterances and the scoring. The scoring process involved detecting disfluencies (restarts, corrections, hesitations, breaks) and determining the types of uttered sentences (e.g., transitive, intransitive, truncated passive). Based on these transcriptions, the onsets and offsets of the word segments were determined with the help of the forced alignment tool provided by the Bavarian Archive for Speech Signals (BAS) [72]. The automatic annotation of speech segments was manually checked at the word level. During this second manual analysis of the speech data, the time stamps of the syntactic constituents (sentential subject, sentential object, finite verb) were also added to the data. All this information was created and stored for each utterance in the TextGrid format provided by Praat. Most importantly, the start of an utterance serves as a measure of the latency in the onset of speech. The boundaries of the first words constitute a critical window for the extraction of the intonation peaks at the beginning of an utterance.

The F0 of each sentence (in Hertz) was extracted with the help of the autocorrelation method available in Praat in two passes (this method is established in [73]). During the first pass, F0 tracks were extracted with default settings for the lowest and highest F0, the “floor” and “ceiling” (75 Hz and 600 Hz, respectively). Then, the first and third quartiles of F0 (Q1 and Q3) were calculated for each speaker and recorded in a table. In the second pass, F0 contours were extracted with speaker-specific settings (0.75*Q1 for floor and 1.5*Q3 for ceiling). The F0 samples making up a contour were saved in a two-column format where the first column contains the timestamps and the second column the F0 measures at the given times (the so-called Pitch Objects).

In addition to TextGrids and Pitch Objects, a spreadsheet was created to check the scoring and store information about the types of noun phrases that were used for the names of the agent and patient. Importantly, the corrected scoring of the utterances (syntactic structure, e.g., transitive, intransitive, truncated passive) was also updated in the TextGrids. Thus, the TextGrids alone constitute a rich linguistic database of these spontaneously produced pictures, as they contain the accurate linguistic analyses of utterances, checked for the segmentation at the word level and prosodic information about the pausing and hesitations.

The timing and position of the eye fixations were recorded relative to the regions of the depicted actors, the so-called areas of interest (AOI). The AOIs were determined on the basis of their thematic roles. This means that the actors sharing a thematic role were enclosed within a single AOI. The AOI was determined independently of the actual eye fixations to cover the agents and patients, and a margin of 100 pixels around them [74]. All fixations were registered as falling within the interest area of the agent, the patient or empty when falling outside the designated AOIs.

Deviating from the pre-registration of the study, the results were included in the analysis only when a participant was able to remember at least one word correctly in 50% of the load trials. This correction had to be made because, all in all, the memory task combined with the speech production task turned out to be rather difficult. The percentage remembering at least one word on the load trials (32) by participant (N = 84) varied between 56 and 75 (the 25% and 75% percentiles). The maximum percentage of remembering at least one word on the load trials was 97%. Adherence to the original inclusion criterion would have left no data for the analysis. In total, 64 study subjects met the updated inclusion criterion. This exclusion based on the memory performance affected 24% of the data.

In addition to excluding participants based on their performance in the memory task, a set of exclusion criteria determined the selection of individual items for the final analysis. The methodology of relating the timing of eye gaze with cognitive processes in sentence planning relies heavily on the timing of the speech and the syntactic structure of the spoken sentences. Therefore, the target trials were excluded when the first fixation to the AOI occured later than 400 ms after the onset of the presentation of the picture, and when the time delay between two consecutive fixations was longer than 600 ms (indication of loss of tracking). The exclusions based on the eye movements affected 2.5% of the data. Furthermore, only fluent transitive sentences were included. Additional trials were excluded based on speech onset latencies longer than three standard deviations away from the grand mean. The exclusions based on sentential structure and speech timing affected 31% of the data. Finally, it was planned to remove all data from the subject when the subject’s inclusion rate after the indicated exclusions was lower than 25%. None of the 64 study subjects met this criterion and their results were all included as long as their utterances satisfied the timing and linguistic requirements. No treatment of missing data was necessary because the modern methods of hierarchical regression modelling (LMER, Bayesian regression modelling; [75–78] respectively) are robust to unbalanced data. Altogether, 52% of the recorded data could be included in the final analyses (see Table 1).

**Table 1:** The number of included utterances as a function of identity (Same, Different), double actor (Agent, Patient) and load (Low, High).

		Same	Different
Load low	Agent	345	317
	Patient	368	333
Load high	Agent	360	314
	Patient	371	362

#### Dependent variables

For the assessment of pre-speech language processing, a new relative measure was derived based on the concept of gaze and current understanding of eye movements in linguistic task settings [16–21, 49, 62, 63]. As outlined in the Methods section, sentence planning research using the visual world paradigm has established that an increased number of consecutive eye fixations directed towards the first-mentioned actor in a picture indicates the lexical retrieval and possibly phonological processing of the actor’s name. Under this definition, these accumulating eye fixations form a longest eye gaze, called the ‘naming gaze’. These naming gazes can be extracted from each trial and evaluated individually, without the need for averaging across study subjects or items.

The naming gaze serves as a landmark for the language processing in the time window between the onset of the presentation of the picture and the start of an utterance. Namely, the time before the onset of the naming gaze corresponds to the time window where the conceptual frame of an incipient utterance is generated. The time after this longest gaze indicates encoding of further aspects of the subsequent utterance (possibly the verb or second-mentioned actor) and typically contains the start of the utterance. The highly incremental processing entails that after the onset of the presentation of a picture, speakers start to encode the first-mentioned actors as soon as possible [18, 26, 62]. Since they might also start their speech early, they may spend most of the time period between the onset of a picture and the start of an utterance with processing the first-mentioned actors. In other words, the increment of conceptual planning is small, corresponding only to the first-mentioned agent role. Conversely, weakly incremental planning entails that speakers take time for processing the second-mentioned actors in addition to the first-mentioned actor. As a result, the naming gaze at the first-mentioned actor starts later [16, 19] and the onset of speech may be delayed compared to the highly incremental planning. In other words, the increment of conceptual planning is large, including both the agent and patient roles and the event.

To capture the small and large planning increments, we devised a ‘measure of relative duration of the naming gaze’. For this, the duration of the naming gaze was divided by the corresponding latency of speech onset. In other words, the duration of the naming gaze measured proportionally to the latency of speech onset constitutes the measure of the relative duration of the naming gaze (for another study measuring the proportion of the duration of a particular gaze, see [30]). Assuming that the absolute duration of the naming gaze remains relatively constant (as observed in the data published in [36]), the expected relative duration of the naming gaze is comparatively large for small increments of conceptual planning and comparatively small for large increments of conceptual planning. Accordingly, the degree of incrementality and the effect of cognitive load on conceptual processing can be inferred from the relative duration of the naming gaze such that the increasing relative duration of the naming gaze at the first-mentioned actor or object approximates the increase of incrementality and a processing delay.

**Fig 5:**
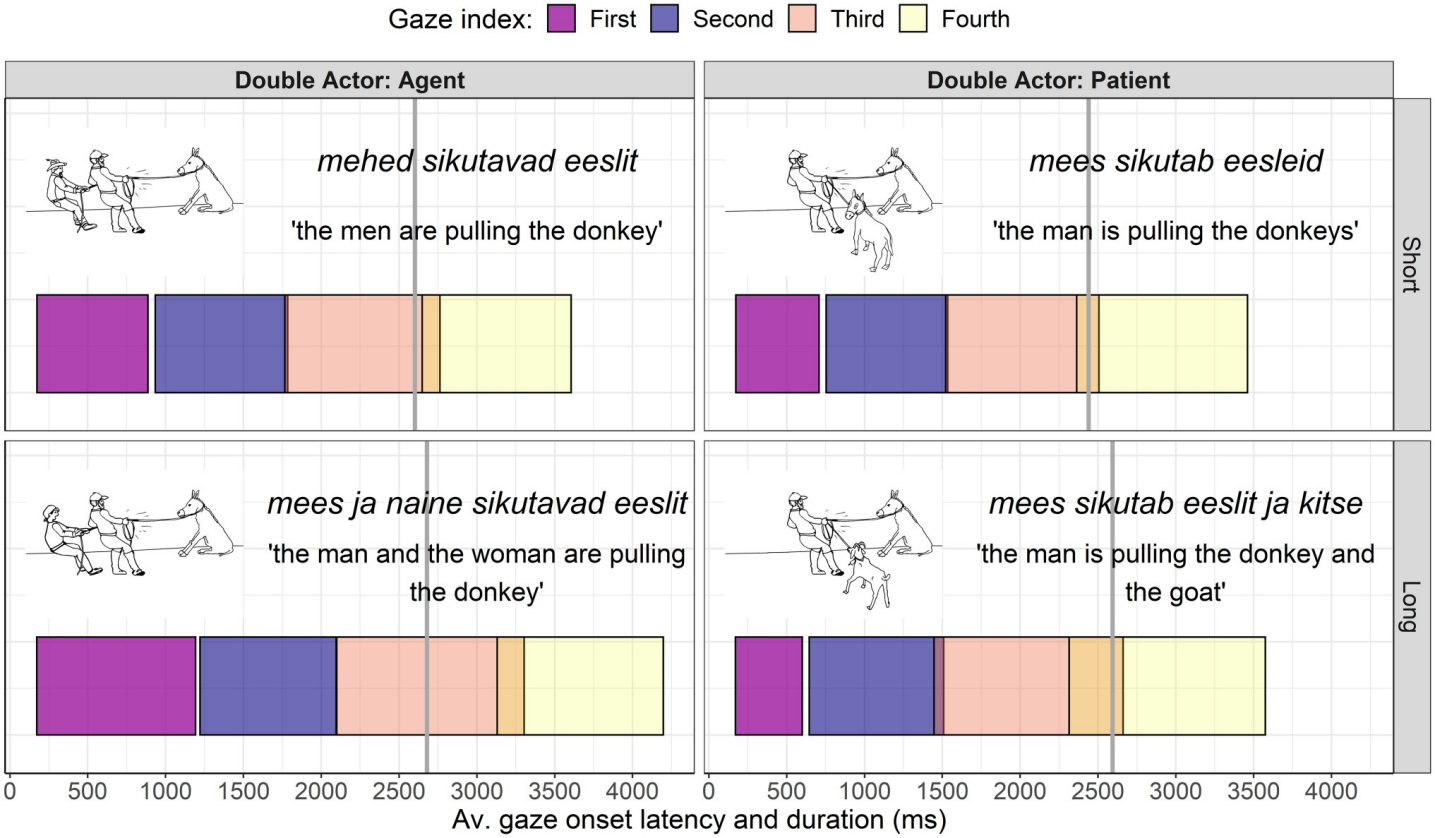
Average start times and average durations of gazes that occurred before the average latencies of the speech onset (grey vertical lines) by their indices. Since the squares represent the average duration of the gazes, they overlap (in a way not possible in individual trials), especially the squares between the third and fourth gazes. Based on the averages, the third gaze (red square) appears across the four conditions as being the longest one and can be taken as illustrating the gaze of longest duration occurring before the onset of speech, the so-called *naming gaze*. In reality, the naming gazes are determined individually for each trial and they do not depend on the gaze index. For assessing the degree of incrementality, the duration of the naming gaze is divided by the latency of the onset of speech, to yield a proportional duration of the naming gaze.

The exploration of the eye movement indicated that the visual processing before the speech onset was spread between one to five gazes (see Fig 5). The number of gazes increased together with the complexity of the visual scenes. The durations of the gazes were calculated by subtracting the onset time of the first fixation directed at the AOI from the offset time of the last consecutive fixation directed to the AOI. Then, among those gazes occurring before the onset of speech, the gaze of maximum duration was detected and assigned as being *the naming gaze*. The naming gaze was reported as missing (NA) when the algorithm could not detect a gaze of maximum duration. Finally, for the relative duration of the naming gaze, the durations of the naming gazes were divided by the respective latencies of the speech onset.

**Fig 6:**
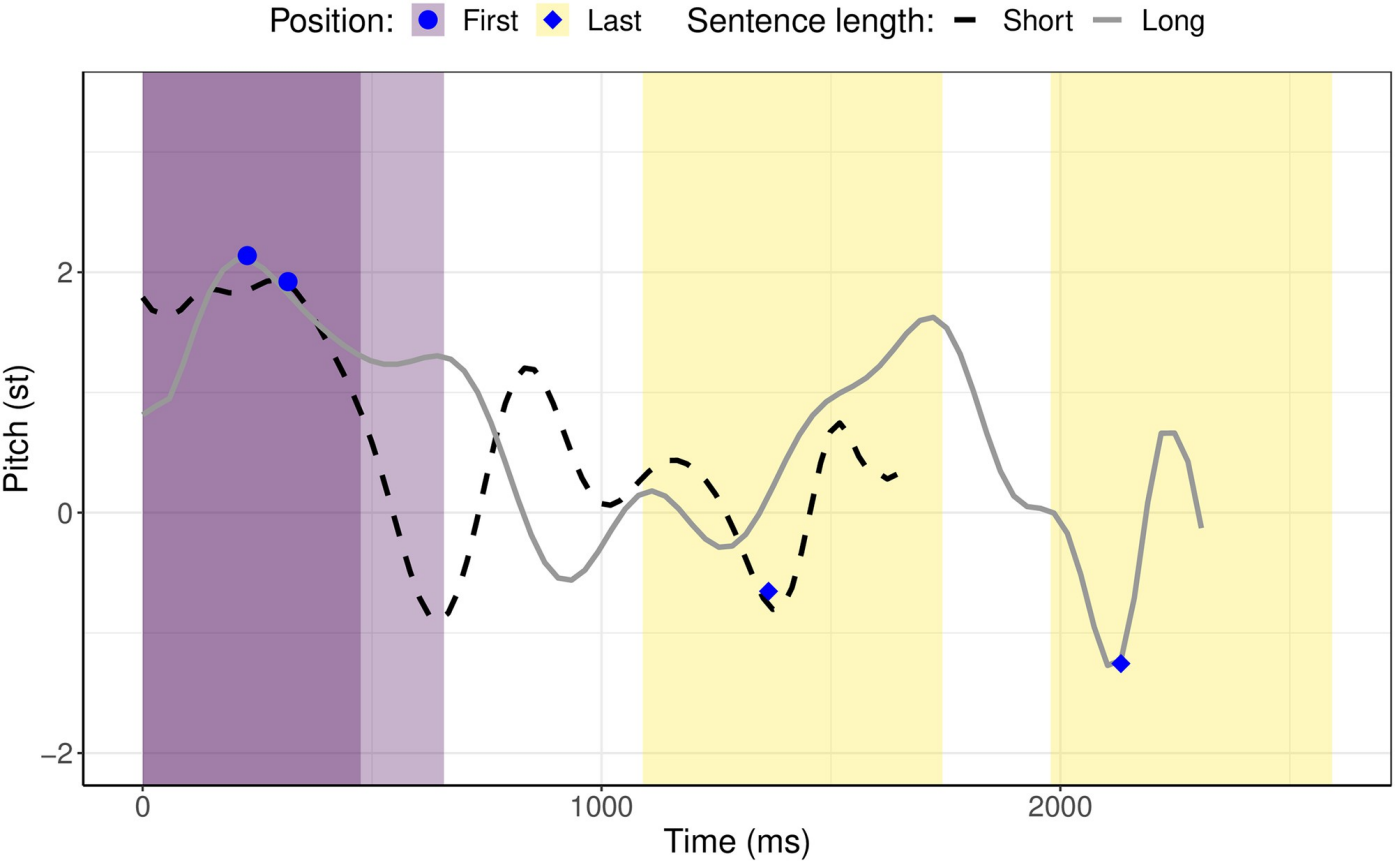
Pitch contours of a sample of short and long utterances spoken in spontaneous Estonian. For illustrative purposes, the lines are polynomial smoothings of degree 22, regressed to pitch values as a function of time. The blue circles indicate the F0 maxima from the words at the beginning of an utterance and the blue diamonds indicate the F0 minima from those at the end of an utterance.

For the examination of intonation planning, the maxima and minima of the F0 were automatically extracted within the boundaries of the words at the beginning of the utterance and those at the end of the utterance (the names of the agent and patient, respectively; see Fig 6). To normalize for gender differences (male speakers typically speak in a lower voice than female speakers do), the maxima and minima of the F0, measured in Hertz (Hz), were converted into semitones (the conversion to semitones was established in [79]) relative to the speaker’s mean, based on Eq (1)

$$\begin{array}{c} F0_{st}=12\times {\text{log}}_{2}(\frac{F0_{\text{Hz}}}{F0_{\text{mean}}}), \end{array}$$ (1)

where *F*0_mean_ is the subject’s mean F0 aggregated over all F0 contours extracted from each of the subject’s utterances. Without controlling for the large-scale, gender-based differences in voice pitch, a potentially smaller length effect on initial intonation peaks might remain concealed.

#### Statistical evaluation

**Fig 7:**
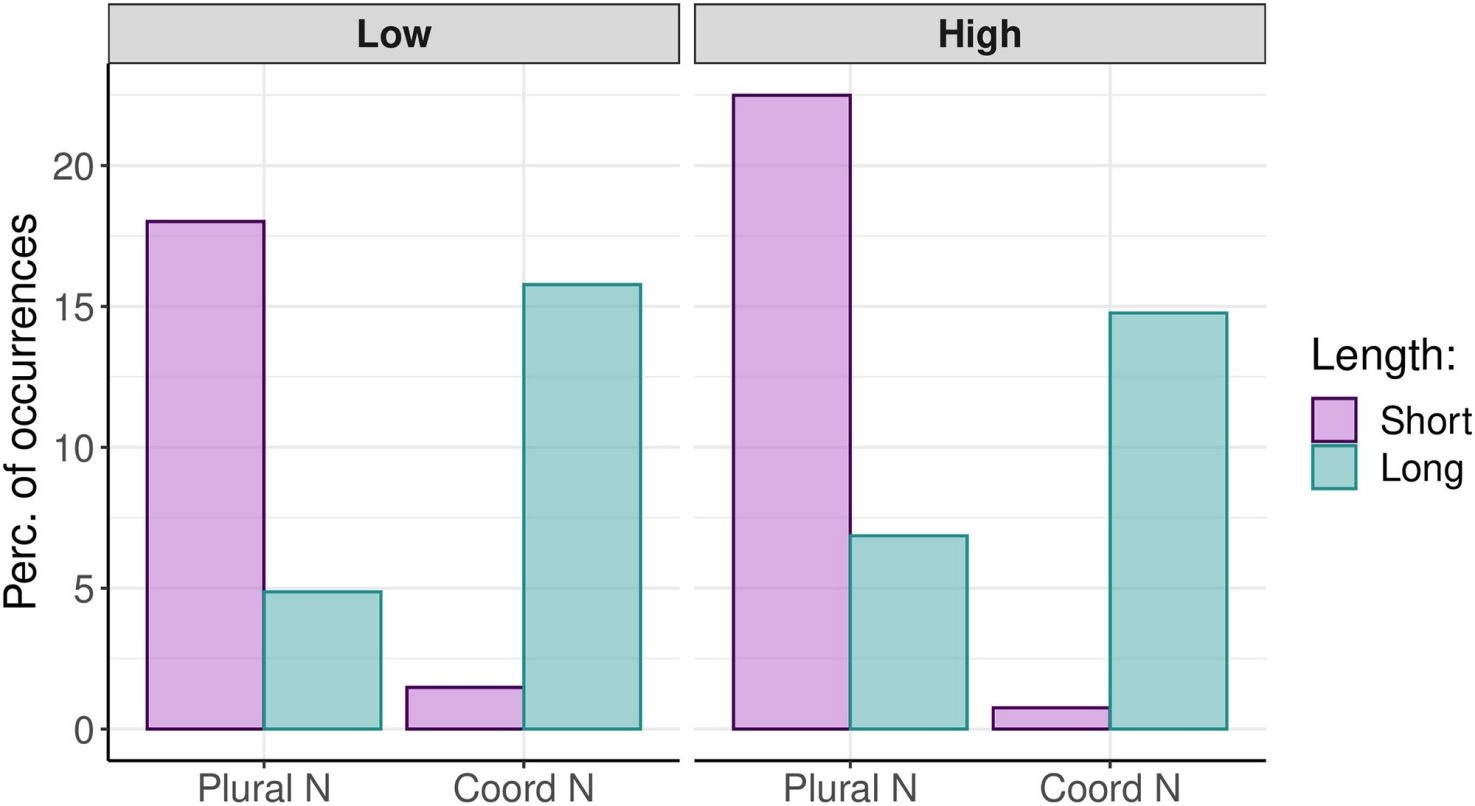
Rate of plural and coordinated noun phrases in short and long utterances under low and high cognitive loads.

For the statistical analysis, the visual factors Double Actor and Identity were reinterpreted with reference to the linguistic forms of the descriptions of the picture to more closely reflect the sentence planning processes. The visual factor Identity accounted for the variation of length of the utterance, with the same actors eliciting short utterances and different actors eliciting long utterances. Depending on the number of the actors sharing the agent or patient roles and the identities of the double actors, the agents and patients were encoded as singular nouns (e.g. Santa), plural nouns (e.g. Santas) or coordinated nouns (e.g. The Santa and the elf; see Fig 7 for the frequency of the plural and coordinated noun phrases). The plural and coordinated nouns constituted the so-called complex constituents. As both the agents and patients could be encoded either as plural nouns or coordinated nouns, the complex constituents in agent–patient utterances occurred either at the beginning of an utterance or at the end of an utterance, which yields a factor, ‘position’. The goal of the statistical inference was to determine whether the factors position, length, and load contribute to the variation of eye gaze and F0 in semi-spontaneous speech. The results are interpreted in terms of the incrementality of the sentence planning, shared resources between language production and WM, and of advance planning of sentence intonation.

The pre-registration of the study defined two sets of analyses. The primary analysis aimed to assess the impact of a two-way interaction between length and load on four dependent variables: (i) relative duration of naming gazes, (ii) absolute duration of naming gazes, (iii) F0 minima at the end of an utterance, and (iv) F0 maxima at the beginning of an utterance. The secondary exploratory analysis aimed to investigate the effect of position in a three-way interaction between position, length, and load on two selected dependent variables: (i) relative duration of naming gazes and (ii) intonation peaks at the beginning of an utterance. All these analyses involved 6 linear mixed effects regression models (LMERs) separated by the different sets of dependent variables and of fixed effects, implemented using the *lme4* package in the R language for statistical computing [75, 80].

While the pre-registration outlined methods for addressing convergence problems following the suggestions provided by [75], subsequent evaluation revealed persistent convergence problems across all types of random effects, particularly those exhibiting complexity. Only the intercept models demonstrated convergence. To preserve the required complexity of the random effects structure, evaluation procedures were switched to Bayesian regression modelling, facilitated by the R software package *brms* [78]. The adoption of Bayesian regression modelling was additionally justified by the observation of relatively small effects of experimental factors in the results. In cases of small effects, the sampling procedures inherent in Bayesian modelling provide increased confidence in discerning a true effect.

Consequently, the four dependent variables (relative duration of the naming gazes, absolute duration of the naming gazes, F0 minima at the end of an utterance, F0 maxima at the beginning of an utterance) were submitted to four separate Bayesian regression analyses for the primary analysis. All regression models included the interaction between the sum-coded factors load (Low vs High) and sentence length (Short vs Long) as the fixed effect. In addition, the maximal random-effects structure allowed the predictors and their interaction to vary by participants and items, as it is justified by the experimental design. The number of the trial and the number of the block were pre-registered as co-variates for the LMER analyses. However, the pre-registered experimental design did not support the analysis of these block and trial numbers as co-variates. The trials with load were always presented in the second block (as was fixed in the protocol above). Therefore, the number of the block (first vs second) was fully correlated with the factor cognitive load (low vs high) and was thus excluded from the analysis to avoid autocorrelation in the regression coefficients. Similarly, the number of the trial was also somewhat correlated with the manipulation of the cognitive load in two blocks because the trials with a cognitive load always had a number higher than 96. Thus, the treatment of the trial number as a co-variate also yielded regression coefficients with autocorrelations, and it was therefore inserted into the model as a semi-random effect with intercepts and slopes varying by item and participant. The inclusion in this way of the number of the trial appeared to improve the Bayesian fit of the data.

The secondary analysis inspected the effect of the three-way interaction between the sum-coded factors, position, length, and load, on the dependent variables, relative duration of the naming gaze and F0 maxima at the beginning of an utterance. Additionally, a third dependent variable (established in the section called “Follow-up analysis” below), the ratio of the delay in the onset of the gaze to the delay in the onset of speech, was included in the exploratory analysis. The three Bayesian regression analyses modeled a dependent variable (relative duration of the naming gaze, F0 maxima at the beginning of an utterance, gaze to speech onset ratio) as a function of the three-way interaction between the position, length, and load, allowing the intercepts and slopes of the fixed effects, their interaction and number of the trial to vary by participant and item, as was justified by the experimental design.

For the measurements of the eye gaze, the Bayesian beta models were computed to take into account their right-skewed and zero bound nature. Weakly informative priors were set for the shape and scale parameters (*α* = 0.2, *β* = 0.2) of the gamma distribution. For the measurements of the F0, Bayesian skewed Gaussian models were computed with a highly skeptical prior for a mean of 2 semitones (*σ* = 4).

Four sampling chains were run for 10,000 iterations with a warm-up period of 5,000 iterations and a thinning rate of 5, resulting in 12,000 samples for each tuple of parameters. The means and 95% highest-density intervals (HDI) are presented for each combination of factor levels and differences between them, with inferences drawn based on posterior probabilities. Probabilities greater than zero are considered as compelling evidence supporting the hypotheses given that zero is not included in the 95% HDI of the estimate and the posterior probability exceeds 0.95.

## Results and discussion

In the following, the first section examines the effect of the two-way interaction between the length and load on the four dependent variables assessing the cognitive processes of sentence planning (relative and absolute duration of the naming gaze) and intonation planning (F0 minima and maxima). The second section presents the results of the impact of the three-way interaction between the position, length and load on the relative durations of the gaze and the F0 maxima.

For the contingency of an increase in the initial intonation peaks on the cognitive demands of planning longer utterances, the relative duration of the naming gaze (indicative of conceptual planning) should increase under a high cognitive load relative to that under a low cognitive load and the F0 maxima in long utterances should be lower under a high load than under a low load.

### Effects of length and load on utterance intonation

The aim of the two-way interaction analyses was to confirm the effect of cognitive load on the incrementality of the conceptual planning (the variable of relative duration of the naming gaze) and on the planning of intonation (the variable of F0 maxima at the beginning of an utterance). The examination of two additional variables, absolute duration of the naming gaze and F0 minima at the end of an utterance provide deeper insights into the planning of sentences and sentence intonation. In particular, the absolute duration of the gaze assesses the linguistic encoding processes and aims to detect an inteference effect during the retrieval of agent names immediately before the onset of an utterance. For the physiological constraints, the F0 minima are expected to be consistent across the different lengths of the utterances.

**Fig 8:**
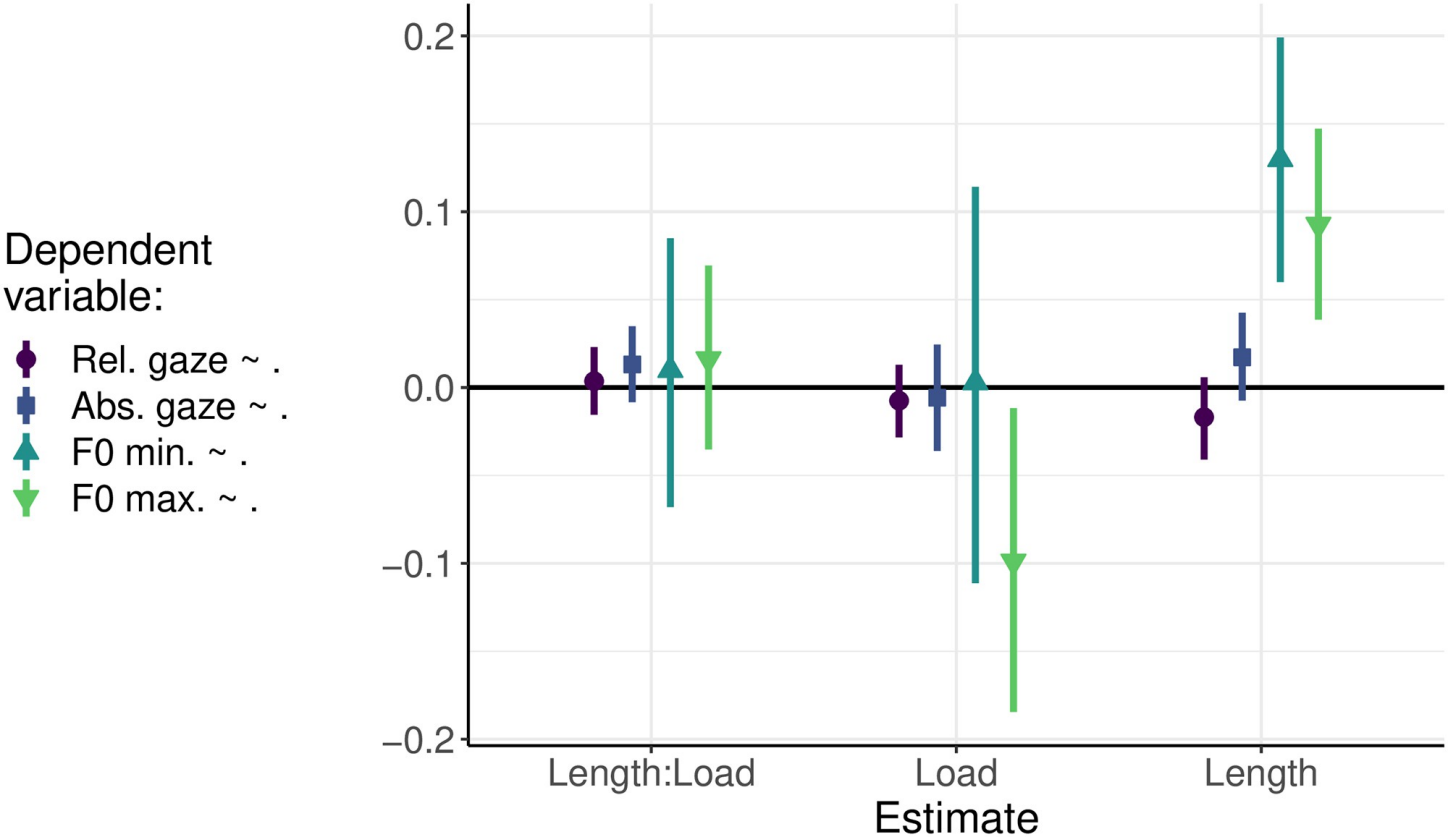
The influence of sentence length (Length) and cognitive load (Load) on four dependent variables (relative gaze duration (logarithm), absolute gaze duration (logarithm), F0 minimum at the end of an utterance (semitones), and F0 maximum at the beginning of an utterance (semitones)) estimated by Bayesian regression analysis. The model terms (Length, Load and the interaction between them) estimate the deviation from the global mean that was in the intercept (not plotted for reasons of clarity). The points and lines indicate estimated means and their 95% confidence intervals. The confidence intervals not touching the zero-line suggest that there is a significant effect of a factor on the corresponding dependent variable.

The results of the Bayesian regression modelling of the four dependent variables are presented in Fig 8. The 95% confidence intervals of the parameters of the model suggest that the measurements of eye gaze remained largely unaffected by variations of the length of the utterance and the cognitive load. In particular, no significant differences were observed between the different conditions of length or load, nor was there a significant interaction between these two factors. However, Fig 8 demonstrates that both measurements of F0 were influenced by the length of the utterance, with F0 maxima also exhibiting a sensitivity to the cognitive load. For the sake of economising on space, the following solely concentrates on the significant effects of length on intonation valleys and length and load on initial intonation peaks.

#### F0 minima at the end of an utterance

Contrary to the assumptions based on the existing literature [8], which suggested that the low pitch range remains consistent across different types of utterances within a speaker, the present study reveals that the intonation valleys are higher in long utterances than in short utterances. Analysis of the posterior densities of the parameter values indicates that F0 minima in short sentences ($E(\mu_{short})=-0.13$, CI=[-0.36–0.087]) are lower than those in long sentences ($E(\mu_{long})=0.13$, CI=[-0.098–0.36]). Moreover, the posterior probability (*P*(*σ* > 0) = 0.9995) of the difference in the F0 between short and long sentences ($E(\mu_{short}-\mu_{long})=0.26$, CI=[0.12–0.4]) provides strong evidence that F0 valleys occur at higher pitches in longer utterances than in shorter ones.

#### F0 maxima at the beginning of an utterance

**Fig 9:**
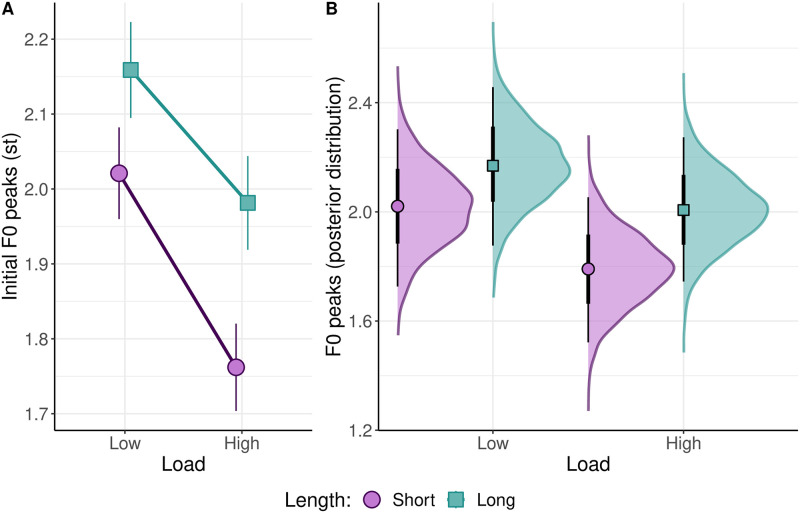
True means and 95% confidence intervals of F0 maxima as a function of length and load (A) and the posterior distributions of means of F0 maxima as a function of length and load with 95% credible intervals (B).

The results revealed a clear impact of the length of the utterance on the height of the intonation peaks at the beginning of an utterance, irrespective of the cognitive load condition. Specifically, the Bayesian regression analysis indicated a significant effect of length on F0 maxima. A posterior density analysis of the parameter values shows that F0 maxima at the beginning of short utterances ($E(\mu_{short})=1.90$, CI=[1.64–2.15], see Fig 9A and 9B) are lower than those at the beginning of long utterances ($E(\mu_{long})=2.09$, CI=[1.84–2.35]). The posterior probability (*P*(*σ* > 0) = 0.9998) of this difference in the F0 between short and long utterances ($E(\mu_{short}-\mu_{long})=0.19$, CI=[0.09–0.26]) provides strong evidence that F0 peaks are higher in longer utterances than in shorter ones. Additionally, F0 peaks under low load conditions are higher than those under high load conditions, regardless of the length of the utterance. Specifically, the posterior density analysis suggests that F0 peaks are higher under a low load ($E(\mu_{low})=2.10$, CI=[1.82–2.37]) than under a high load ($E(\mu_{high})=1.90$, CI=[1.65–2.15]). The posterior probability (*P*(*σ* > 0) = 0.986) of this difference in the F0 between low and high load conditions ($E(\mu_{low}-\mu_{high})=0.2$, CI = 0.02–0.37]) provides compelling evidence that F0 peaks are lower under a high cognitive load than under a low cognitive load.

Altogether, the results demonstrate the effect of length on sentence intonation under both high and low cognitive loads. Thus, speakers were able to plan the intonation of a sentence despite the high cost of the speaking task. However, the restrictions on the capacity of the WM yielded lower F0 peaks at the beginning of utterances regardless of the length of the utterance. Consequently, the results indicate a sensitivity of sentence intonation to restricted WM capacity.

Unexpectedly, the eye movements failed to indicate increasing incrementality in the stage of message generation. This surprising absence of a load effect on sentence processing might be explained by the well-known notion of memory decay, which is especially known to affect phonological information (see, e.g., [81]). Therefore, as the time progressed, more of the language-related working memory buffers might have become available through the decay of items in the word list. Subsequently, more cognitive capacity could be allocated to the description of the picture and an effective planning of the utterance, leading to an absence of any effect of the limitations on the capacity of WM.

Intonation valleys, as indexed by F0 minima extracted from words at the end of an utterance, varied with the length of the utterance. This finding contradicts the physiological account, which predicts a consistently low register for utterances of varying lengths [6, 8, 10]. However, a similar length-dependent variation in final intonation valleys were observed in an earlier study of Estonian [13], where it was argued that the variation was caused by the incomplete nature of the pause-delimited prosodic chunks. These chunks could have been parts of intonation phrases or entire utterances, but did not necessarily constitute an entire utterance. It was concluded, thus, that the incomplete nature of these pause-delimited prosodic chunks led to the varying intonation valleys.

Here, intonation valleys were measured from final positions defined by the content of the utterance. An initial boundary of an utterance coincided with the start of the agent name, and the final boundary included the mention of the patient name. The intonation valleys had a higher pitch in longer utterances than they did in shorter utterances. This may suggest that longer utterances were segmented into several prosodic phrases, with the pitch level adjusted to a higher register at the beginning of each non-initial prosodic chunk. In longer utterances, the prosodic chunking might have counteracted the declining trend of the pitch that began with the very first pitch accent. The grey F0 contour in Fig 6 at approximately 1750 ms seems to confirm this suggestion. Thus, as the pitch levels were reset over the course of longer utterances, the pitch valleys had a higher levels than in shorter utterances.

Alternatively, the higher intonation valleys might reflect rising trends of the intonation at the ends of longer utterances. More complex visual scenes could have induced uncertainty in the speakers, leading them to avoid committing to their statements by employing rising intonation contours at the ends of their utterances. This behavior is indicated by the earlier findings that associate rising pitch accents with a lack of the speaker’s commitment (see [82]). Consequently, the rising intonation contours might cause the F0 minimum extracted from the last word of the utterance to be scaled at a higher pitch level than the actual low register. A much more detailed intonation analysis is necessary to confirm this hypothesis. Such an analysis goes beyond the scope of the present study and is left for future research.

### Effect of position on planning sentences

The exploratory analyses in this section examine the effects of a three-way interaction between position, length, and cognitive load on the relative duration of the naming gaze and F0 maxima at the beginning of an utterance. As before, the relative duration of the naming gaze indexes incremetality of conceptual planning and F0 maxima approximate planning for sentence intonation.

**Fig 10:**
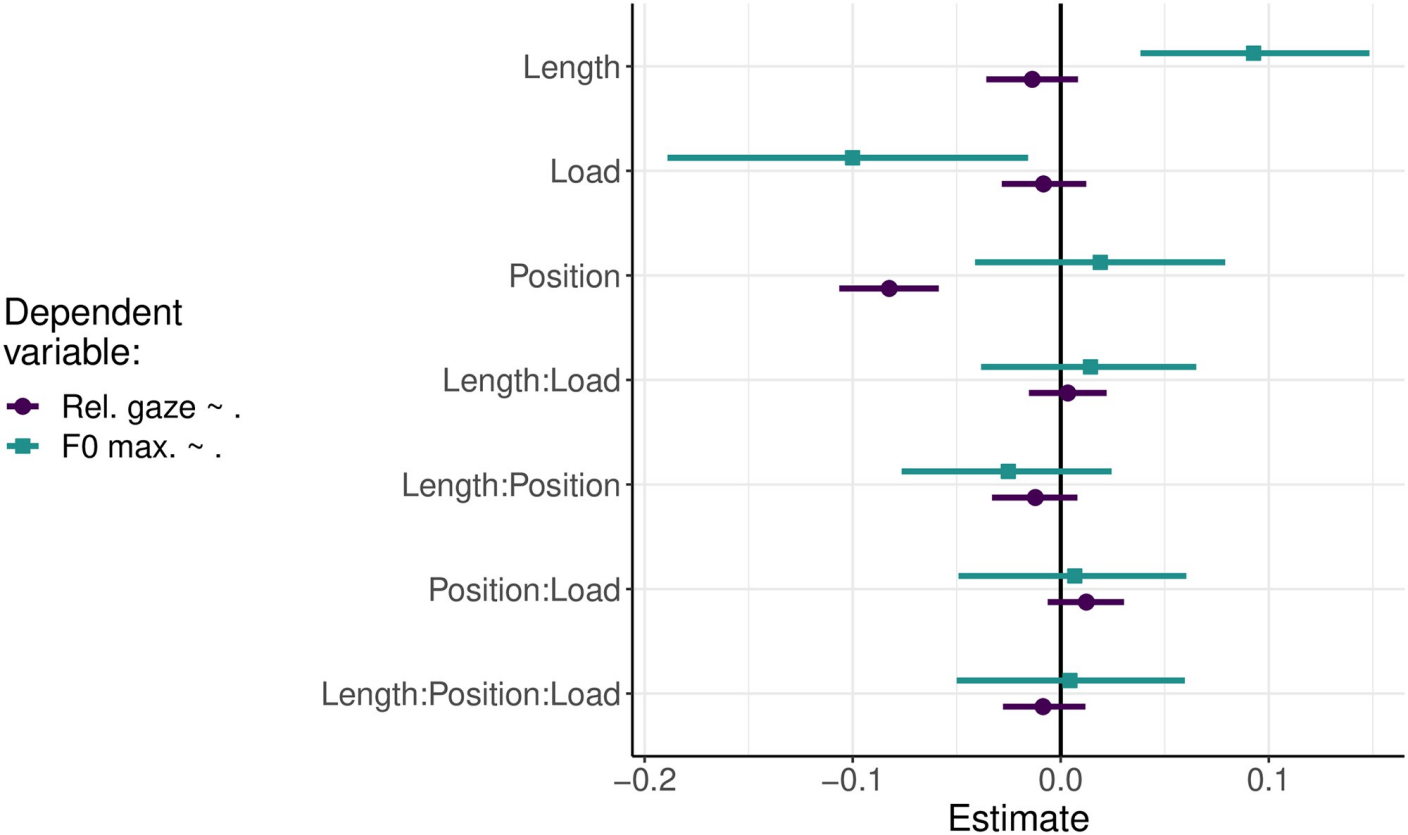
The influence of sentence length (Length), cognitive load (Load) and position (Position) on two dependent variables (relative gaze duration (logarithm) and F0 maxima at the beginning of an utterance (semitones)) estimated by Bayesian regression analysis. The model terms (Length, Load, Position and the interactions between them) estimate the deviation from the global mean that was in the intercept (not plotted for the reasons of clarity). The points indicate estimated means and the lines their 95% confidence intervals. The confidence intervals not touching the zero-line suggest that there is a significant effect of a factor on the corresponding dependent variable.

The confidence intervals in Fig 10 of the values of the parameters derived from the Bayesian regression analysis demonstrate that the relative duration of the naming gaze was influenced by the position of the complex constituent, while remaining unaffected by the length and load factors, as well as their interactions. Additionally, the results suggest that intonation peaks at the beginning of an utterance were not influenced by the position of the complex constituent or by interactions between the position, length, and load. However, they were found to be sensitive to manipulations of the length and load, consistent with previous two-way interaction analyses. In the following, we will concentrate only on the significant effects.

#### Relative duration of the naming gazes

**Fig 11:**
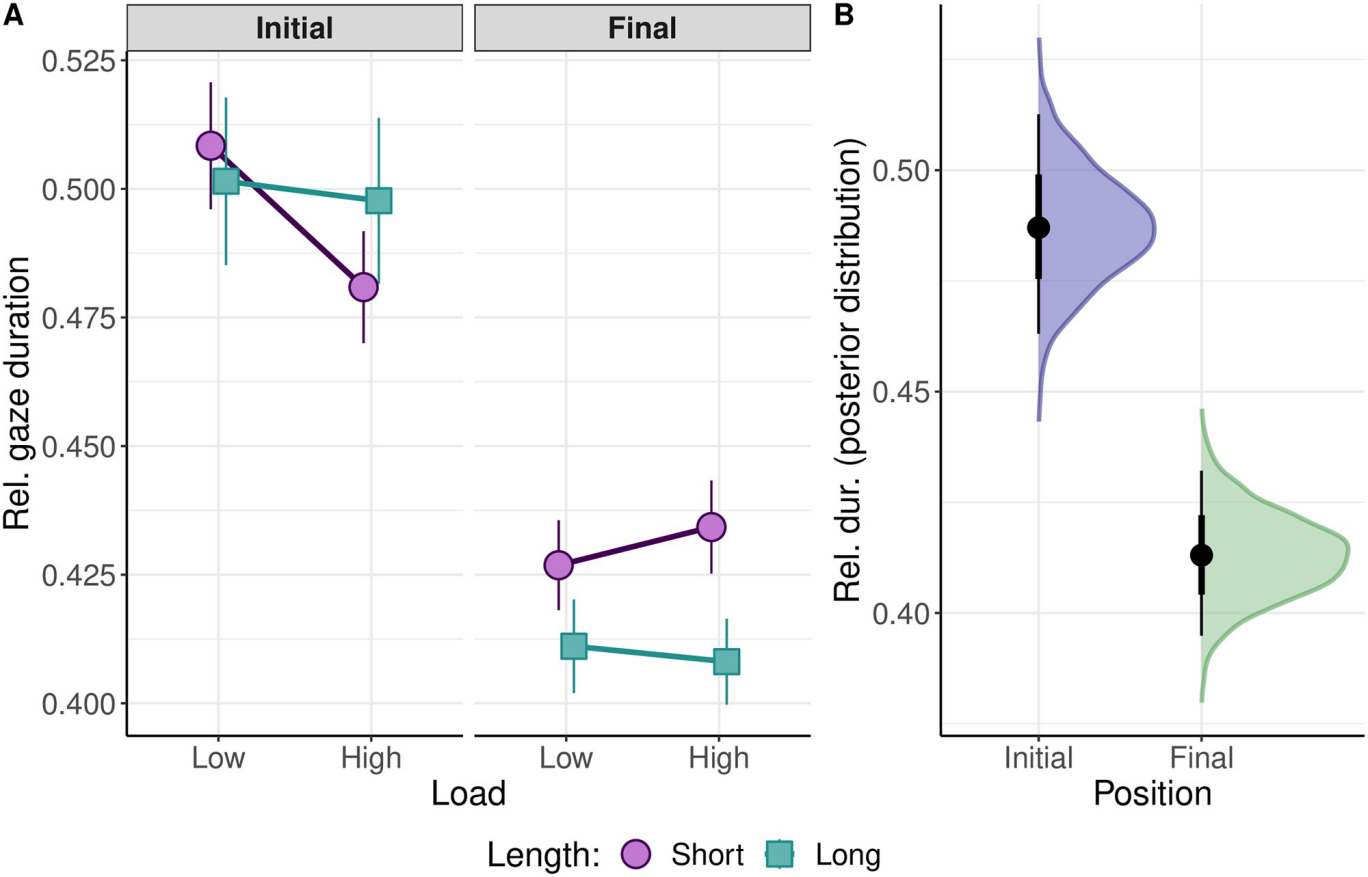
True means and 95% confidence intervals of the relative gaze duration (A) and the posterior distributions of the means of the relative gaze durations with 95% credible intervals (B).

The relative duration of naming gazes was found to vary with position. The posterior densities of the parameter values revealed that naming gazes were longer for the complex constituents in the initial position ($E(\mu_{initial})=0.49$, CI=[0.46–0.51]; see also the results and the posterior densities of parameter values in Fig 11A and 11B) compared to those in the final position ($E(\mu_{final})=0.41$, CI=[0.4–0.43]). The posterior probability (*P*(*σ* > 0) = 0.9954) of this durational difference between initial and final positions ($E(\mu_{initial}-\mu_{final})=0.07$, CI=[0.05–0.1]) provides compelling evidence that naming gazes are longer for complex constituents mentioned at the beginning of an utterance than for those mentioned at the end.

#### F0 maxima at the beginning of an utterance

The three-way interaction analysis of F0 maxima yielded results similar to the two-way interaction analysis. The posterior densities of the parameter values indicated that F0 maxima at the beginning of short utterances were lower ($E(\mu_{short})=1.90$, CI=[1.63–2.15]) than for those in long utterances ($E(\mu_{long})=2.09$, CI=[1.83–2.36]). The posterior probability (*P*(*σ* > 0) = 0.9988) of this difference in F0 between short and long sentences ($E(\mu_{short}-\mu_{long})=0.19$, CI=[0.08–0.24]) provides compelling evidence that F0 peaks are higher in longer utterances. Additionally, F0 peaks were higher under a low load ($E(\mu_{low})=2.10$, CI=[1.83–2.39]) than under a high load ($E(\mu_{high})=1.90$, CI=[1.65–2.17]). The posterior probability (*P*(*σ* > 0) = 0.9898) of this difference in F0 between low and high loads ($E(\mu_{low}-\mu_{high})=0.2$, CI=[0.03–0.38]) provides compelling evidence that F0 peaks are lower under a high cognitive load.

Overall, the results underscore the effect of length on sentence intonation in Estonian, regardless of the sentential position of the complex constituent. This result runs counter to the expectation provided by the existing study on read-aloud speech in German [4]. The insensitivity of initial intonation peaks to sentential position found in the present study indicates a extensive planning increment for sentence intonation, as the scaling of the intonation peaks for NPs at the end of a sentence did not differ from those for NPs at the beginning of a sentence.

Moreover, the present study reveals the sensitivity of eye movements to the visual arrangement of the actors, with longer relative durations of naming gazes observed for double actors mentioned at the beginning of utterances compared to those mentioned at the end. Notably, the posterior means for the initial and final placement of the double actors are 49% and 41%, respectively, suggesting that visual and linguistic attention was allocated to the third single actor in the visual display for the other half of the initial picture processing time. This distribution of visual attention across the three actors indicates an extensive planning increment for conceptual processing. Related to this, the complexity of the scenes and the speaking task may have contributed to the selection of a non-incremental planning strategy by the speakers in this study. Nonetheless, the relative duration of the gaze successfully detects a small 8% variation in the scope of the planning. Given the stringent control over visual arrangements, this variation reliably suggests differing planning scopes for messages, particularly evident in the smaller conceptual planning scope evoked by mentioning of complex constituents at the beginning of an utterance.

Furthermore, the overall non-incremental planning strategy aligns with the large planning scope of sentence intonation. It is important that the two sentential positions of the complex constituent did not lead to aligned scopes of sentence planning and intonation planning, suggesting that intonation planning may not be directly related to the scope of message planning. However, pitch peaks exhibited considerable variation, which is characteristic as they are influenced by numerous linguistic and extralinguistic factors and the experiment was restricted to controlling for the between-item pitch variation. The 8% change in the scope of message planning might have been too subtle for average intonation peaks to reliably respond to this varying degree of incremantality. Therefore, the results remain inconclusive with regard to the relation between the processes of sentence planning and of intonation planning.

### Follow-up analysis: Effect of load on conceptual processing

The goal of the follow-up analysis was to further examine the timeline of picture and sentence processing. While the relative and absolute durations of the naming gazes were found to be insensitive to the manipulations of cognitive load, another analysis aimed to determine whether the onset of the naming gaze exhibits interference effects from the two simultaneous linguistic tasks. As pictures were presented immediately after the memorization of the three nouns, the outset of picture processing should not be affected by severe memory decay. Therefore, we decided to explore the interval between the onset of a picture and the onset of the naming gaze. To normalize again for variations in the onsets of utterances in spontaneous speech, the delay of the naming gaze from the onset of a picture was divided by the corresponding delay of the speech onset, resulting in a ‘gaze-to-speech ratio.’ Consistent with the known interference effects, the gaze-to-speech ratio is expected to increase as the memory load on the speaker increases. Thus, a larger gaze-to-speech ratio indicates a processing delay.

**Fig 12:**
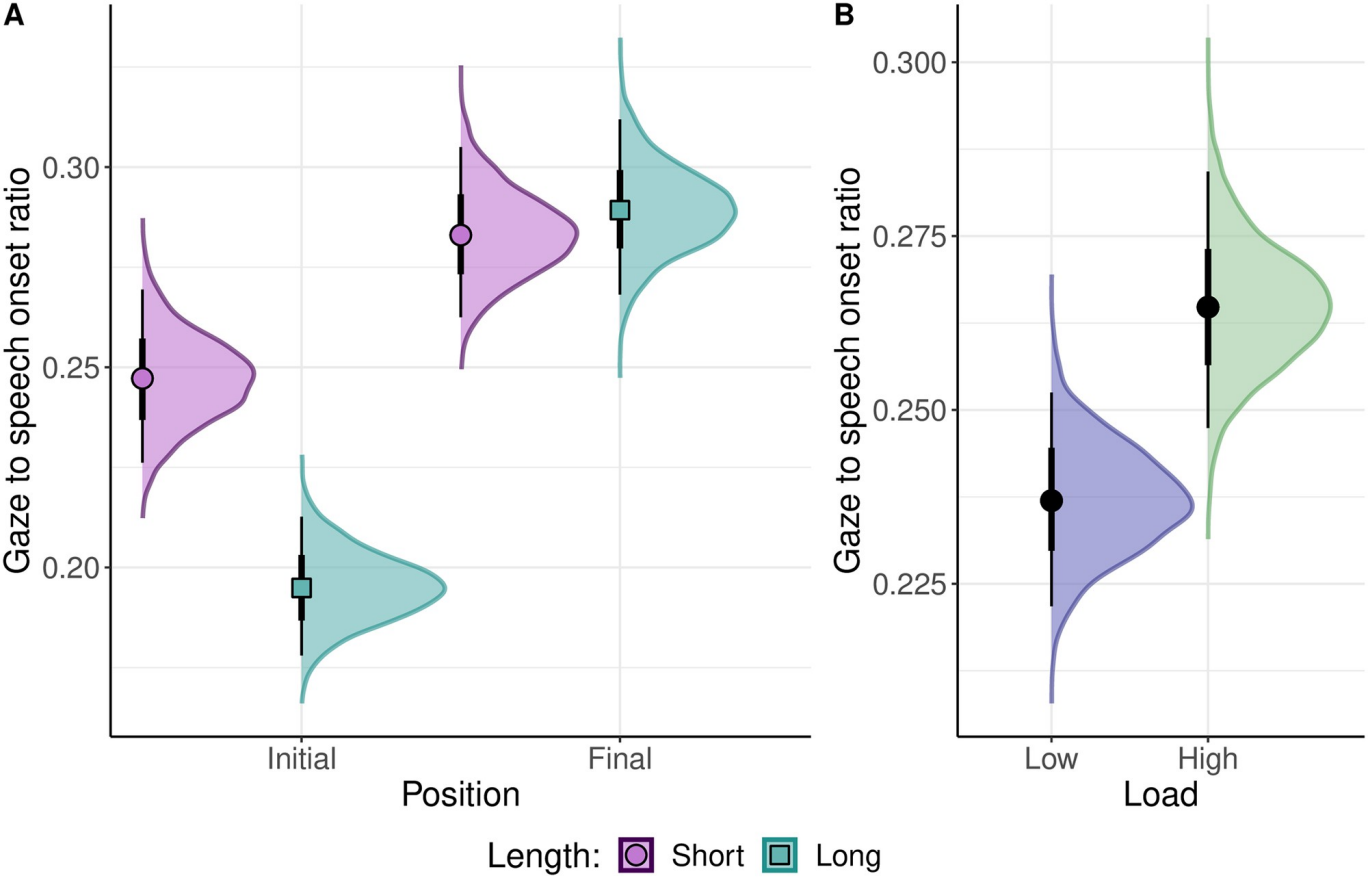
The posterior samples of the means of the ratio of the delay in the onset of the naming gaze to the delay in the onset of speech as a function of position and utterance length (A) and of load (B), with lines indicating the 95% credible intervals.

The posterior densities of the means obtained from the Bayesian regression modelling are illustrated in Fig 12. The results reveal a high sensitivity of the gaze-to-speech ratio to the factors of length, load, and position. The Bayesian regression modelling of the posterior samples uncovered a significant two-way interaction between length and position on the gaze-to-speech ratio.

For the complex constituents at the beginning of an utterance, the posterior densities of the values of the parameters indicate that the gaze-to-speech ratio in a short utterance is greater ($E(\mu_{initial,short})=0.25$, CI=[0.23–0.27]) than in a long utterance ($E(\mu_{initial,long})=0.2$, CI=[0.18–0.21]). The posterior probability (*P*(*σ* > 0) = 1) of this timing difference of the naming gaze between shorter and longer utterances ($E(\mu_{initial,short}-\mu_{initial,long})=0.05$, CI=[0.03–0.07]) provides compelling evidence that the onset of the naming gaze varies as a function of the length of the utterance when the complex constituent is located at the beginning.

Analysis of the final position of the complex constituent suggests that the gaze-to-speech ratio in a short utterance ($E(\mu_{final,short})=0.28$, CI=[0.26–0.3]) does not differ from that for a long utterance ($E(\mu_{final,long})=0.29$, CI=[0.27–0.31]). The posterior probability (*P*(*σ* > 0) = 0.3088) of this timing difference between shorter and longer utterances ($E(\mu_{final,short}-\mu_{final,long})=-0.01$, CI=[-0.03–0.02]) does not provide any evidence for the onset of the naming gaze to vary as a function of the length when the complex constituent is located at the end of an utterance.

To assess the impact of position as modulated by the interaction term, the analysis compared short utterances containing complex constituents at their beginning to long utterances containing complex constituents at their end. The results indicate that having complex constituents at the end of an utterance elicits greater ratios of the delay in the onset of the naming gaze to the onset of speech ($E(\mu_{final,long})=0.29$, CI=[0.27–0.31]) compared to constituents at the beginning of an utterance ($E(\mu_{initial,short})=0.25$, CI=[0.23–0.27]). The posterior probability (*P*(*σ* > 0) = 1) of this timing difference of the onset of the naming gaze ($E(\mu_{final,long}-\mu_{initial,short})=0.04$, CI=[0.02–0.07]) between initial and final positions provides compelling evidence that the onset of the naming gaze is delayed for the complex constituents occurring at the ends of utterances relative to those occurring at the beginning.

Cognitive load also had an impact on the gaze-to-speech ratio, with this ratio being higher under a high cognitive load ($E(\mu_{high})=0.27$, CI=[0.25–0.28]) than under a low load ($E(\mu_{low})=0.24$, CI=[0.22–0.25]). The posterior probability (*P*(*σ* > 0) = 0.9992) of this timing difference between high and low load ($E(\mu_{high}-\mu_{low})=0.03$, CI=[0.01–0.04]) provides compelling evidence that the gaze-to-speech ratio is greater under high cognitive load than under low cognitive load.

Admittedly, within the context where the relative duration of the naming gaze was argued to be an effective measure of the scope of the conceptual planning stage, the justification of the delay in the onset of the naming gaze, or the gaze-to-speech ratio as another measure of incrementality is somewhat challenging. However, this measure aligns well with the earlier observations that the conceptual planning stage constitutes about 30% of the pre-speech picture processing time [17–19, 62]. For example, the conceptualization stage as indexed by the gaze-to-speech ratio was 25% of the pre-speech picture processing time for the utterances mentioning complex constituents that occurred at the beginning of the sentence (e.g. ‘The Santa and the elf are decorating the Christmas tree’). The gaze-to-speech ratio reflects the relative duration of the naming gaze in that it was dependent on the position of the complex constituent. In addition, it was more sensitive to the factors of sentence length and cognitive load and it might therefore more accurately capture the time point where the earliest conceptual planning stage is completed and the processes of lexical retrieval and linguistic encoding begin.

In particular, the gaze-to-speech ratio revealed a distinction between planning short and long utterances. However, the difference in planning strategies was only observed for the complex constituents at the beginning of utterances. Specifically, the notably shorter ratios for the long complex constituents at the beginning of an utterance mean that the naming gaze started shortly after the onset of a picture. The long gaze-to-speech ratios indicate that the onset of the naming gaze was delayed relative to the onset of a picture. Taking the longer time delays to index the size of the planning increments, the results suggest that the utterances with the complex constituents at the beginning were planned highly incrementally. It seems that for the initial coordinated NPs, speakers initiated speech as soon as they conceptualized the first noun of the coordinated NP, delaying the planning of the remainder of the utterance until the onset of speech. This strategy is well anticipated for long and complex utterances. Curiously, however, utterances with complex noun phrases at their end were planned further ahead.

Moreover, the gaze-to-speech ratio was able to detect a very short-lived interference effect of the word recall task. As anticipated, the onset of the naming gaze was delayed under high cognitive load relative to low cognitive load. This delay suggests that the cognitive system, at the onset of picture presentation, was preoccupied with maintaining the three nouns in the (verbal) WM, thereby disrupting the smooth conceptualization processes of the descriptions of a picture. However, the results of the relative and absolute gaze durations indicate that the interference effect lasted for a very brief period and was entirely overcome by the time of the linguistic encoding of the utterance. This was probably necessitated by the complex task setting and the arrangements of the actors in the visual displays. Possibly, the task complexity may have contributed to the pronounced shift of focus between maintaining the memory items and describing the pictures, leading to the eventual decay of the three nouns. The interference effect during message generation might be better detectable in the descriptions of simpler pictures.

The reason for the relative duration of the naming gaze being less sensitive than the gaze-to-speech ratio may lie in the fact that it evaluates all pre-verbal processing except for the processing of the first-mentioned referent. For example, in a scenario where the processing of agents (i.e., the first-mentioned referents) concludes early before the onset of speech, and the gaze shifts to patients (i.e., the last-mentioned referents), the measure of the relative gaze duration includes extensive conceptual planning but most likely also, some linguistic encoding of patients, which might have commenced before the onset of speech as well. This aspect of the relative measure of the naming gaze could pose a limitation for discerning more subtle cognitive changes during the pre-linguistic processing stage.

## General discussion and the conclusion

The visual world paradigm was employed to observe real-time cognitive processing of language for speaking. An eye-tracking experiment was integrated into a dual-task paradigm, aimed at examining the cognitive resources shared between working memory (WM) and language production. For language production and speech prosody in particular, the results indicated an effect of memory load that disrupted the conceptual processing and influenced the subsequent scaling of initial intonation peaks.

In particular, the study demonstrated the effect of utterance length on initial intonation peaks, a phenomenon observed in numerous previous studies [6, 8, 10, 11, 13, 35, 37]. This length effect underscores the notion of advance planning of sentence intonation, suggesting that speakers anticipate the length of the upcoming utterance and adjust the height of the very first pitch accent according to the length of the utterance. Unexpectedly, intonation valleys varied as a function of sentence length, being higher in longer utterances than in shorter ones. This finding aligns with the existing results for Estonian spontaneous speech (see [13]). As discussed in the Results section, the longer utterances might have been segmented into several prosodic phrases, with the decreasing trend in the F0 resetting to a higher level during the course of longer utterances. Alternatively, the measurement of the final F0 minima could have been influenced by the rising pitch accents. The inconsistency of low level pitch at the ends of utterances indicates that the low pitch register is not as constrained for variation as suggested in earlier studies [6, 8, 10]. Furthermore, the final F0 minima might indicate small phonological planning increments, but this possibility should be explored more closely in future studies.

Despite a length-dependent scaling of final intonation valleys, the average height of intonation peaks in longer utterances was about 10% of the average peak height of shorter utterances. Thus, the variation of low pitch levels did not revoke the length-dependent scaling of pitch at the beginning of an utterance. Thus, the physiological necessity of intonation planning does not hold consistently and an intriguing question arises: what other mechanisms drive the length-dependent F0 scaling? The present study proposed and tested a cognitive account of advance planning of sentence intonation where it was suggested that the larger planning increments necessary for the utterance of long and complex utterances exhaust the verbal WM buffers, which in turn underlies the higher initial intonation peaks in longer utterances compared to shorter ones. In other words, the rise in pitch can be viewed as a tonal response to the heightened mental load.

For the relationship between sentence intonation and the cognitive cost of speaking, the study predicted lower initial intonation peaks under a high cognitive load relative to a low cognitive load and a disappearance of pitch difference between the short and long utterances (see Fig 1). This hypothesis was partly confirmed. In particular, the results showed that initial intonation peaks were lower under a high cognitive load than under a low cognitive load. However, the prediction that the pitch difference between the two types of utterances would disappear was not borne out. In other words, the experiment did not reveal any cognitive constraints on intonation planning.

Relatedly, the relative and absolute durations of the naming gaze indicated that Estonian speakers generally prefer to plan their messages and utterances less incrementally rather than more incrementally. Specifically, pre-speech visual attention was equally distributed among the actors in visual displays, suggesting that the event encoding encompassed all the event participants. The converging measures of eye gaze and F0 suggest that intonation planning might be driven by a comprehensive conceptual framework and extensive lexical retrieval occurring at the interface of message generation and linguistic encoding. More importantly, the extensive pre-planning of utterances and sentence intonation was not compromised by the additional demands of speaking, likely due to the decay of phonological information in WM, simplifying the word recall task. Specifically, based on a third measure of eye gaze (i.e., the gaze-to-speech ratio), it was argued that a brief processing delay occurred at the outset of picture and utterance processing but was overcome by the time of linguistic encoding, which, in turn facilitated the effective intonation planning at the onset of speech. Further experiments with a higher degree of memory load would clarify the fragility of linguistic encoding and intonation planning in the face of a high cognitive cost of speaking.

Does the study nevertheless provide some insight into the lowering effect of cognitive load on intonation peaks? To assess whether pitch elevation at the beginning of an utterance is contingent on language-related WM capacity, an experiment was designed to explore the interference effect known from previous studies [14, 15, 42–45]. This interference effect arises from two similar tasks, such as the recall of linguistic elements and picture naming, compromising each other and resulting in prolonged processing times. Previous studies on interference effects have shown that the scope of phonological planning decreases under a high cognitive load [15, 25]. However, it has been challenging to determine whether the same holds true for conceptual planning and semantico-syntactic planning [14, 15, 45]. This study aimed to address this gap by investigating whether eye movements could serve as an indicator of interference effects in the cognitive processes of message planning and/or lexical retrieval when one linguistic task interferes with another. The relative and absolute duration of the naming gazes was hypothesized to increase due to the shortage of language-related WM buffers. Specifically, the relative duration of the naming gazes was expected to reflect changes in the conceptual planning of incipient utterances, while the absolute duration served as an indicator of changes in lexical retrieval. However, the durational measurements of the naming gaze did not reveal any processing delays dependent on the level of cognitive load.

Instead, it was the ratio between the delay of the naming gaze from the onset of a picture and that of speech, referred to as the gaze-to-speech ratio, that revealed cognitive changes during the message generation stage. The lengthening of the gaze-to-speech ratio indicated that conceptual planning becomes more incremental with longer sentences, especially when more complex constituents (such as plural or coordinated noun phrases) are mentioned at the beginning of an utterance. In addition, and partly mirroring the measure of the relative duration of the naming gaze, the gaze-to-speech ratio effectively highlighted the variation of smaller and larger planning increments, reflecting more or less incremental planning depending on the subsequent linguistic output.

The increasing effect of the gaze-to-speech ratio is somewhat more ambivalent than the increase of the relative duration of the naming gaze because in addition to a less incremental planning strategy, it also turned out to signal the expected interference effect and the processing delay under a high cognitive load. In contrast, the predicted increase in relative duration of the naming gaze is more straightforward: the same degree of lengthening would assess both the disturbance due to WM load and the incrementality of the conceptual planning. However, the additive processing delays originating from the less incremental message generation and cognitive load need to be considered in the case of the gaze-to-speech ratio.

The increase in the gaze-to-speech ratio under a high cognitive load aligned with the lowering effect of the cognitive cost of speaking on the intonation peaks. This lowering effect of a high cognitive load on initial intonation peaks may represent a tonal response to the short-lived interference effect experienced at the beginning of conceptual processing. Although subsequent stages of linguistic encoding and lexical retrieval showed an efficient recovery from the memory load, the sentence intonation still appeared to retain the cognitive stress encountered at the outset of the picture and sentence processing, evident at the time of the onset of speech. Although somewhat indirect, this finding appears to align with the results in [45], where it was observed that an interference effect was present when speakers needed to recall the relative size of an object before the initiation of an utterance, but not when they needed to recall digits. They interpreted this as a phonological interference effect arising at the level of conceptual processing. Similarly, the current findings suggest a delay in conceptual processing due to high cognitive load and a phonetic/phonological effect of lower pitch peaks. Thus, the present results might corroborate the idea of the interference effect at the conceptual level spreading through linguistic processing stages, subsequently reaching the phonological encoding. Alternatively, there might be a direct link between conceptual processing and pitch scaling. These and other aspects of language production and sentence planning could be considered in future studies.

The low intonation peaks under high cognitive load appear to reflect both intonation planning and limitations on mental resources. It remains to be confirmed whether the limitations on memory at the conceptual processing stage also trigger smaller planning increments. Currently, real-time measures of processing incipient utterances in Estonian replicate large planning units (see [36] for earlier findings) that were not reduced under high cognitive load. The delaying effect observed in the gaze-to-speech ratio indexes the deteriorating effect in the working memory but remains ambiguous with respect to the incrementality of the message generation. However, given the finding in [56] that individuals with smaller memory spans exhibited lower intonation peaks, the planning increments in the present study might also have been smaller under a high load than under a low load. More generally, the present study demonstrates that the lower scaling of initial intonation peaks may indicate limited access to internal WM resources (i.e. language-related WM buffers) during the conceptual processing of incipient utterances.

While the study primarily investigates the cognitive mechanisms underlying sentence intonation, it is crucial to consider the potential influence of language-specific characteristics. The observed effects on initial intonation peaks might differ across languages due to inherent linguistic and phonetic properties. For example, Estonian, the language on which our study was based, exhibits a great degree of case-morphological marking of noun phrases and a full declinational paradigm for verbs [83]. Given the existing research on speech planning, these properties motivate an expectation for larger rather than smaller planning increments, especially at the interface of conceptual planning and linguistic formulation [20, 21, 62]. The findings of this and the most recent study on real-time sentence planning in Estonian align well with this expectation but might further indicate that the planning increments in case-morphological languages may be relatively persistent. In addition, Estonian displays relatively consistent intonation patterns. As reported by earlier research, spoken nouns are accompanied by regular falls and rises across an utterance, which decline towards the end [84, 85]. The uniform pattern of pitch accents and the regular pitch decline might well accommodate the planning of pitch. Languages with more extensive intonational variation might behave differently under high cognitive load. Furthermore, languages might differ in their inclination for the size of a planning increment. Consequently, the size of the planning increment might interact with the cognitive resources in the WM and this may show differing effects in pitch scaling. Future research should aim to explore cross-linguistic differences in greater depth, comparing languages with different scopes of planning and diverse phonological systems to understand how language-specific features impact the cognitive processes involved in speech production.

To conclude, the study investigated the relationship between the cognitive demands of speaking and planning intonation based on semi-spontaneous utterances. Employing a dual-task design, the experiment manipulated the extent of cognitive resources available for the sentence planning to determine whether message generation and linguistic encoding in longer utterances requires a greater amount of cognitive resources, which, in turn, potentially contributes to the length-dependent elevation of intonation peaks at the beginning of an utterance. Notably, the pitch difference between long and short utterances persisted through conditions of low and high load, indicating a robust planning of sentence intonation. The measurements of eye movements jointly indicated that the speech production system first encountered some cognitive stress at the level of message generation but recovered from it effectively at the interface of the conceptual and linguistic encoding. Subsequently, by the onset of speech, the greater parts of the incipient utterances might have been activated and the intonation peaks at the beginning of the utterance could be planned according to the length and complexity of the utterance. Nevertheless, intonation peaks were lower under a high cognitive load, indicating the sensitivity of sentence intonation to the processing delay at the level of conceptual processing. Hence, modulations of sentence intonation demonstrate remarkable flexibility and adaptability to the linguistic and cognitive demands of speaking. Consequently, the findings of this study indicate considerable potential for further experiments aimed at manipulating the available cognitive resources to gain deeper insights into the cognitive mechanisms underlying the planning of sentence intonation.
